# Image-based evaluation of single-cell mechanics using deep learning

**DOI:** 10.1186/s13619-025-00239-9

**Published:** 2025-06-05

**Authors:** Zhaozhao Wu, Yiting Feng, Ran Bi, Zhiqiang Liu, Yudi Niu, Yuhong Jin, Wenjing Li, Huijun Chen, Yan Shi, Yanan Du

**Affiliations:** 1 School of Biomedical Engineering, Tsinghua University, Beijing, 100084 China; 2 Tsinghua-Peking Center for Life Sciences, Tsinghua University, Beijing, 100084 China; 3 School of Basic Medical Sciences, Tsinghua University, Beijing, 100084 China

**Keywords:** Deep learning, Convolutional neural network, Biomechanics, Cell stiffness evaluation

## Abstract

Mechanical properties of cells have been proposed as potential biophysical markers for cell phenotypes and functions since they are vital for maintaining biological activities. However, current approaches used to measure single-cell mechanics suffer from low throughput, high technical complexity, and stringent equipment requirements, which cannot satisfy the demand for large-scale cell sample testing. In this study, we proposed to evaluate cell stiffness at the single-cell level using deep learning. The image-based deep learning models could non-invasively predict the stiffness ranges of mesenchymal stem cells (MSCs) and macrophages in situ with high throughput and high sensitivity. We further applied the models to evaluate MSC functions including senescence, stemness, and immunomodulatory capacity as well as macrophage diversity in phenotypes and functions. Our image-based deep learning models provide potential techniques and perspectives for cell-based mechanobiology research and clinical translation.

**Supplementary Information:**

The online version contains supplementary material available at 10.1186/s13619-025-00239-9.

## Background

Cells possess particular mechanical properties that respond to external mechanical stimuli in vivo. The inherent mechanical properties of cells are necessary to maintain their physiological functions, and changes in these mechanical properties can reflect phenotypic and functional alterations within pathological processes (Kawauchi et al. 2017; Weaver 2017). In particular, cell stiffness is an integrative parameter summarizing the biophysical outcome of many known and unknown biological processes. For example: cancer cells are significantly softer than normal cells, facilitating metastasis and invasion during tumorigenesis (Alibert et al. 2017); resistant cells possess higher stiffness than responsive cells in anti-cancer drug screening (Islam et al. 2018); and mesenchymal stem cells (MSCs), the promising therapeutic candidates for regenerative medicine, undergo replicative senescence during in vitro culture expansion accompanied by a significant increase in cell stiffness (Szydlak et al. 2019; Yang et al. 2018). MSC stiffness can also reflect differentiation potential. Softer MSCs prefer to differentiate into adipocytes while stiffer MSCs prefer to differentiate into osteocytes (Mu et al. 2020; Szydlak et al. 2019). Moreover, macrophage stiffness significantly increases after classical activation or being cultured on rigid substrates. Changes in macrophage stiffness correlate with their inflammatory responses including TNF-$\alpha$ release (Patel et al. 2012). Therefore, cell stiffness can potentially serve as a label-free biophysical marker for stem cell potency and macrophage status.

However, current methods for measuring the mechanical properties of individual cells suffer from low throughput, usually at the rate of tens of cells per hour. Atomic force microscopy (AFM) is the widely-accepted approach used to measure cellular stiffness at the single-cell or subcellular levels. Cells are first compressed using AFM probes with known spring constants, then measured by lasers to detect deformation status. Young’s modulus, which quantifies the ratio between said compression and deformation, can be obtained by fitting the force-indentation curve with the Hertz model (Hao et al. 2020; Wu et al. 2018). Other methods, such as optical tweezers and micropipette pipetting, have also been used to measure the mechanical properties of individual cells by applying external forces and detecting the corresponding cellular responses(Ayala et al. 2016; González-Bermúdez et al. 2019; Hao et al. 2020). However, those methods usually demand high-cost facilities, significant technical expertise, and cannot meet the general requirements for large-scale cell testing due to low throughput. To counter these challenges, high-throughput, microfluidics-based methods have been developed in recent years. Although cell stiffness can be efficiently extracted by detecting cell deformation in microfluidics using micro-constriction or flow-based methods (Lange et al. 2015; Mietke et al. 2015; Nyberg et al. 2017; Voronin et al. 2020) these methods do not allow for *in-situ* single-cell analysis without removing the cells from their microenvironment.

**Fig. 1:**
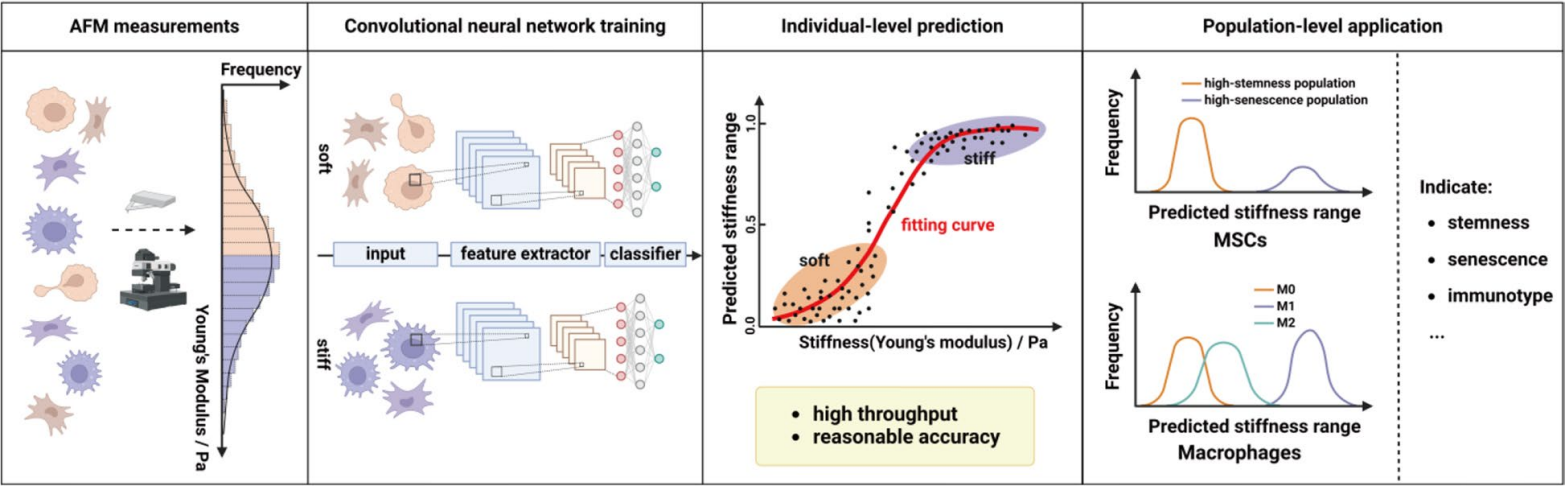
Schematic representation for the image-based cell stiffness evaluation using deep learning. The classification-based models were established to evaluate the range of cell stiffness and applied to explore the correlations between cell stiffness and functions of MSCs and macrophages

To address the challenges in cell stiffness evaluation, we proposed using deep learning tools to analyze cell images (Fig. 1). As the shape of a cell depends primarily on cellular structures, surface tensions, and external forces, cellular morphology can potentially serve as an indicator for cell mechanics (Lecuit and Lenne 2007). Recent studies have shown that softer cancer cells have larger nuclei and more irregular shapes (Wu et al. 2020), while softer macrophages cultured on soft substrates exhibit greater roundness (Patel et al. 2012; Rostam et al. 2017). Similarly, embryonic stem cells with varying Young’s modulus display differences in circularity and diameter (Bongiorno et al. 2018), and MSCs show changes in cortical shear modulus depending on cell volume and substrate stiffness (Guo et al. 2017). Therefore, we believe that morphological differences can indicate variations in cell stiffness. On the other hand, deep learning-based cell image analysis has emerged as a useful tool for various biomedical applications. Notably, deep learning has shown promise in predicting MSC differentiation lineages and efficiency using just bright-field images (Hassanlou et al. 2019; Matsuoka et al. 2013). Similarly, CNN models have outperformed traditional biomarkers in predicting hematopoietic and neural stem cell lineages (Buggenthin et al. 2017; Zhu et al. 2021). Additionally, deep learning can distinguish normal from senescent endothelial cells and assess their senescence degree (Kusumoto et al. 2021). It can also differentiate activated effector T cells from naive T cells using autofluorescence intensity images with high accuracy (Wang et al. 2020). These examples highlight deep learning’s potential in analyzing cell images for various tasks.

We applied deep learning to evaluate single-cell mechanical properties using bright-field cell images. We first established stiffness classification models to distinguish soft and stiff subpopulations of MSCs and RAW264.7 cells, a murine macrophage cell line. We then modified these models to predict intermediate cell stiffness by mapping the probability of a cell being classified as stiff to its actual stiffness level. To validate the models, we compared them with AFM and deformability cytometry (DC), two widely used methods. The modified models were also used to assess stemness, senescence, and immunomodulatory capacity of MSCs from different batches, as well as to identify stiffness differences between pro-inflammatory M1 and pro-healing M2 macrophages. Using a limited dataset of single-cell images from MSCs, RAW264.7 cells, and LSECs labeled with AFM measurements, we developed a deep learning-based stiffness regression model across cell types. This model was applied to explore mechanical responses of MSCs, RAW264.7 cells, and LSECs to varying substrate stiffness. Our work provides proof-of-concept evidence for image-based cell stiffness evaluation using deep learning.

## Results

### Image-based evaluation of MSC stiffness ranges using a deep learning-based stiffness classification model

**Fig. 2:**
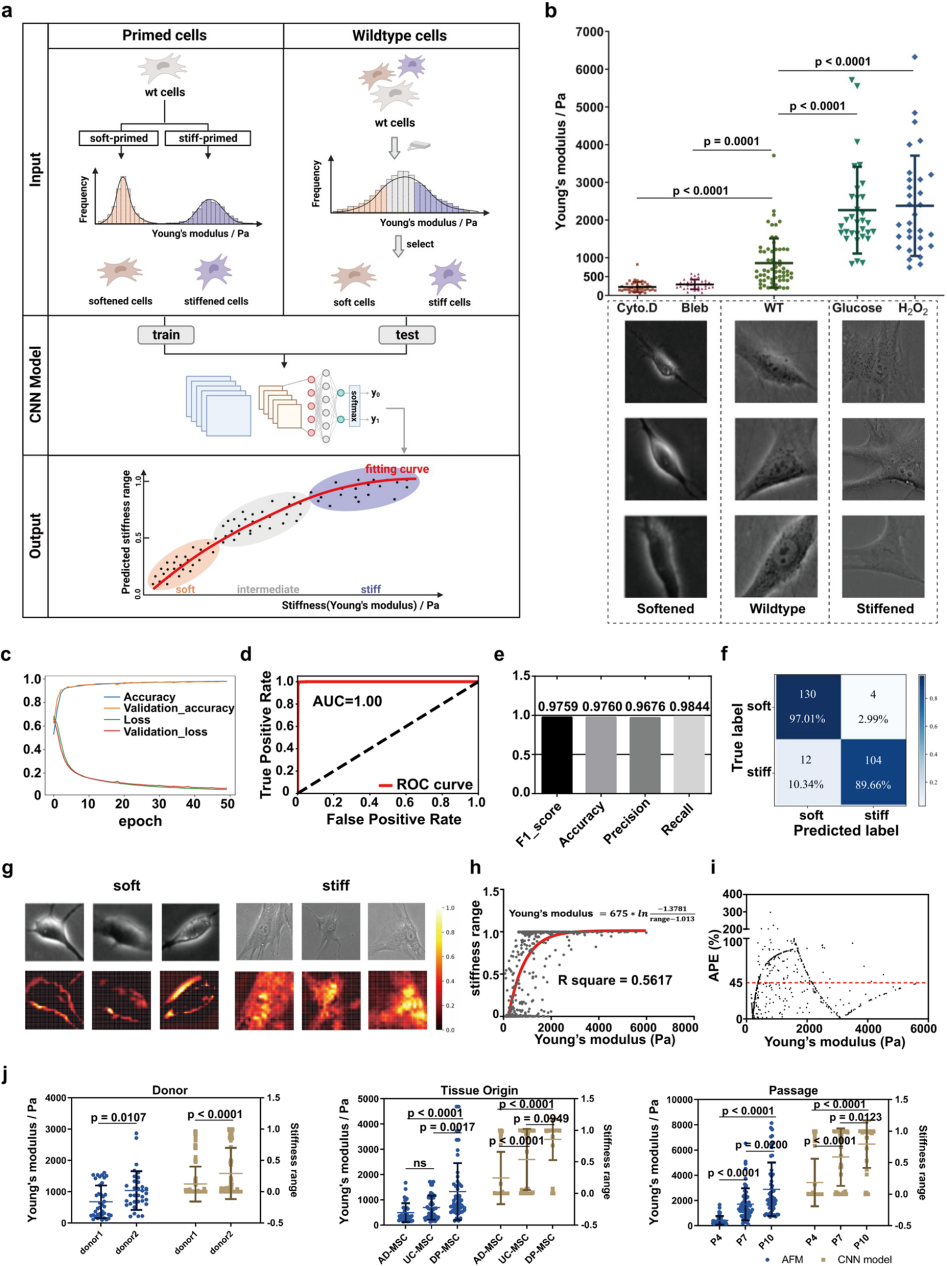
Image-based evaluation of MSC stiffness ranges using the MSC stiffness classification model. **a.** Schematic for the establishment of the MSC stiffness classification model. **b.** Young’s modulus of MSC subpopulations measured with AFM. Soft MSC subpopulations were obtained after treatment with 5 $\mu$ M Cyto.D for 0.5 h or 25 $\mu$ M Bleb for 2 h; stiff subpopulations were treated with 25 mM glucose or 0.15 mM H_2_O_2_ for 5 days. **c.** The learning curve for the stiffness classification model. **d-e.** Indexes for the classification performance on the test set including the AUC under the ROC, the F1_score, accuracy, precision, and recall. **f.** Confusion matrix for the classification performance on wt MSCs with low and high stiffness measured with AFM. **g.** Grad-CAM shows the important regions that influence classification decisions. **h.** The curve for Young’s modulus measured with AFM and corresponding predictive stiffness ranges using the stiffness classification model. The red line represents the one-phase decay fitting. **i.** The APEs between Young’s modulus measured with AFM and predictive Young’s modulus converted from stiffness ranges. The red dot line indicates the mean. **j.** Evaluation of MSC batches from different donors, tissue origins and passages using AFM and the stiffness classification model respectively. AD-MSC adipose tissue-derived MSCs, UC-MSC umbilical cord-derived MSCs, DP-MSC dental pulp-derived MSCs. n ≥ 3 per group. Statistical analysis was performed using Mann–Whitney U-test and Kruskal–Wallis test. Results are presented as mean ± SD

To evaluate the feasibility of stiffness evaluation using deep learning-based classification models, we chose MSCs as our primary cell type. We collected single-cell images from both soft and stiff subpopulations of MSCs and used them to train a deep learning-based classification model. The model was specifically designed to classify MSCs based on their stiffness. We then tested the model’s ability to evaluate the intermediate stiffness of wild-type (wt) MSCs (Fig. 2a). AFM was used to measure the Young's modulus of cells by fitting the force-indentation curve with the Hertz model. Training deep learning models such as CNNs requires a sufficiently large dataset of soft and stiff cell subpopulations, which cannot be easily obtained by low-throughput AFM measurements. To build such a dataset, we generated an abundant number of soft and stiff cells primed by different compounds. To obtain the softened MSC subpopulations, we treated MSCs with Cytochalasin D (Cyto.D) and Blebbistatin (Bleb)(Liu et al. 2017; Mu et al. 2020), resulting in a Young’s modulus predominantly within the range of 200 ± 100 Pa. Conversely, we induced stiffening in MSCs by treating them with glucose and H_2_O_2 _(Wilhelm et al. 2014; Yang et al. 2011), resulting in a Young’s modulus ranging within 3 ± 1 kPa. The differently-primed MSCs effectively encompassed the stiffness range typically observed in in vitro cultured and expanded wt MSCs, where the Young’s modulus commonly falls between 200 Pa and 3 kPa (Fig. 2b), making them suitable for building our dataset. We also tested other approaches, such as osmotic pressure or actin polymerization promoter Jasplakinolide (Jasp)(Mu et al. 2020), to modulate MSC stiffness. However, those methods did not change MSC stiffness significantly compared with wt MSC subpopulations (Fig. S1).

**Fig. 3:**
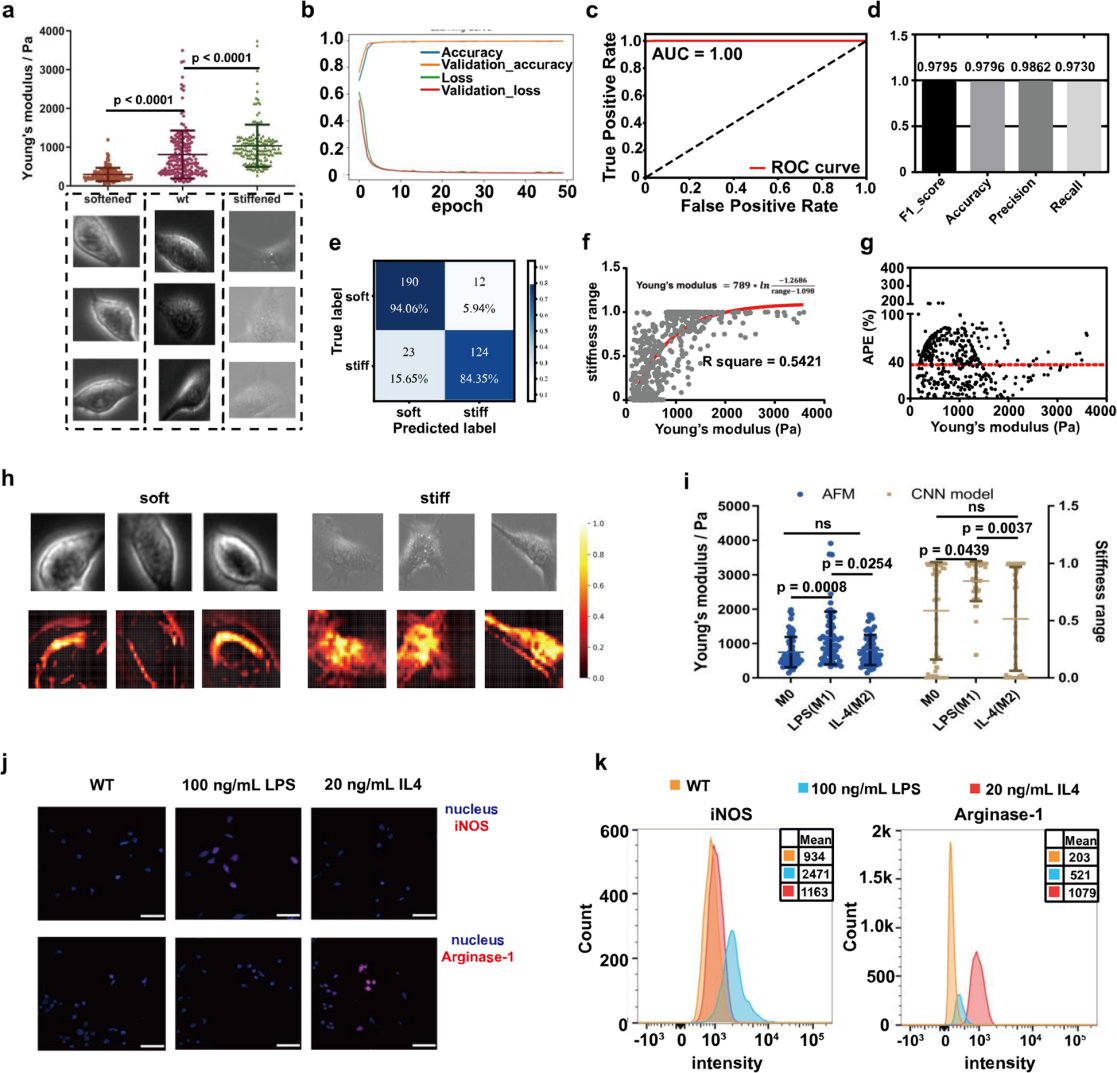
Image-based evaluation of macrophage stiffness ranges using the RAW264.7 stiffness classification model. **a.** Young’s modulus of RAW264.7 subpopulations measured with AFM. Soft RAW264.7 subpopulations were obtained after treatment with 10 $\mu$ M Cyto.D for 2 h and stiff subpopulations were treated with 0.5 mM H_2_O_2_ for 5 days. **b.** The learning curve for the stiffness classification model. **c-d.** Indexes for the classification performance on the test set including the AUC under the ROC, the F1_score, accuracy, precision, and recall. **e.** Confusion matrix for the classification performance on wt RAW264.7 with low and high stiffness measured with AFM. **f.** The curve for Young’s modulus measured with AFM and corresponding predictive stiffness ranges. The red line represents the one-phase decay fitting. **g.** The APEs between Young’s modulus measured with AFM and predictive Young’s modulus converted from stiffness ranges. The red dot line indicates the mean. **h.** Grad-CAM shows the important regions that influence predictions. **i.** Stiffness evaluation of macrophages treated with 100 ng/mL LPS or 20 ng/mL IL-4 for 24 h. **j-k.** Macrophage phenotype characterization using iNOS (M1)/Arginase-1 (M2) in IF and FACS. n ≥ 3 per group. Statistical analysis was performed using the Kruskal–Wallis test. Results are presented as mean ± SD

We then captured phase-contrast images of the softened and stiffened MSC subpopulations. After applying preprocessing techniques, we obtained a total of 60,210 single-cell images for the softened MSCs and 60,126 single-cell images for the stiffened MSCs (Fig. S2). The dataset was split into training (60%), validation (20%), and test sets (20%). We developed a CNN-based stiffness classification model (Fig. 2c), comparing its learning curves with classical models like AlexNet (Fig. 3a), VGG16 (Fig. S3b), Inception V3 (Fig. S3c), and ResNet-50 (Fig. S3d). Our CNN model, with a simpler structure and fewer parameters, achieved high accuracy (> 0.9) without overfitting. Compared with these classical classification models, our CNN-based model had a simple network structure and a small number of parameters, which reduced the cost of model training and optimization. Evaluated on the test set, it showed an AUC of 1.00, F1-score of 0.9759, accuracy of 0.9760, precision of 0.9676, and recall of 0.9844 (Fig. 2d, e). When applied to wt MSCs (200 ± 100 Pa as soft, ≥ 3 kPa as stiff), the model achieved true positive rates of 0.9701 for soft and 0.8966 for stiff MSCs (Fig. 2f). Using Grad-CAM (Selvaraju et al. 2020), we visualized that the model classifies MSCs by recognizing bright peripheral regions in soft cells and heterogeneous intracellular regions in stiff cells (Fig. 2g).

We evaluated the CNN-based classification model’s ability to assess intermediate stiffness in wt MSCs. In line with prior research (Wang et al. 2020), we applied class probability to stiff MSCs (ranging from 0 to 1), generated by the softmax activation function in the final layer of our CNN, for quantifying the stiffness range of individual MSCs. The higher the stiffness range, the greater the MSC stiffness. Thus, this MSC stiffness classification model can be utilized to output a quantitative estimation of the cell stiffness of individual MSCs. We correlated AFM-measured Young’s modulus with stiffness ranges predicted by the model, fitting the data using a one-phase decay function (Fig. 2h). The mean absolute percentage error (APE) between AFM measurements and model predictions was 45% (Fig. 2i). while the mean coefficient of variation for AFM measurements on MSCs was 15% in this work (Fig. S4a) and previous research showed that the APEs of AFM measurements at populated levels fell within 15% ~ 100% (Wu et al. 2018). At the populated level, we obtained MSC batches from different donors, tissue origins, and passages cultured in vitro. We evaluated MSC stiffness by the MSC stiffness classification model and AFM in parallel. Consistent with AFM measurements, the CNN-based classification model identified the stiffness differences among MSCs derived from different donors, tissue origins, and passages (Fig. 2j). Overall, this CNN-based classification model could distinguish soft and stiff MSCs with high accuracy. This conclusion highlights the potential of our model to be utilized as a reliable tool for assessing the stiffness characteristics of MSCs in diverse experimental settings.

### Image-based evaluation of macrophage stiffness ranges using a deep learning-based stiffness classification model

Next, we selected the macrophage cell line RAW264.7 to verify the generalizability of evaluating intermediate cell stiffness based on pre-trained stiffness classification models. Similarly, we constructed a deep learning-based classification model trained with single RAW264.7 images within soft and stiff subpopulations, and verified its capacity in evaluating the intermediate stiffness of wt RAW264.7. Through the application of Cyto.D treatment, we successfully obtained a subpopulation of softened RAW264.7 cells with a stiffness range of approximately 200 ± 100 Pa. Conversely, treatment with H_2_O_2_ allowed us to generate a subpopulation of stiffened RAW264.7 cells with a stiffness range of approximately 2 ± 1 kPa. It is worth noting that the majority of untreated RAW264.7 cells exhibited Young’s modulus values within the range of 200 Pa to 2 kPa (Fig. 3a).

To ensure a robust training process, we collected a total of 60,876 phase-contrast single-cell images for the softened RAW264.7 subpopulation and 61,128 single-cell images for the stiffened RAW264.7 subpopulation. Following the same partitioning ratio as in the MSC dataset (6:2:2), we divided these images into separate sets for training, validation, and testing. Next, we constructed the CNN-based classification model trained with the training set and obtained the learning curve of the CNN-based RAW264.7 stiffness classification model (Fig. 3b). This CNN-based classification model performed strongly on the test set. The AUC under the ROC curve was 1.00 and the F1_score, accuracy, precision, and recall were 0.9795, 0.9796, 0.9862, and 0.9730 respectively (Fig. 3c, d). Additionally, we tested the CNN’s performance using wt RAW264.7. Cells with 200 ± 100 Pa Young’s modulus were labeled as soft RAW264.7 and cells with 2 ± 1 kPa or above were classified as stiff RAW264.7. The true positive rates of the CNN-based classification model were 0.9406 for soft RAW264.7 and 0.8435 for stiff RAW264.7 (Fig. 3e). We used the Grad-CAM algorithm to visualize whether there existed universal cell regions that indicated cell stiffness for both MSCs and RAW264.7. Similarly, Grad-CAM indicated that the CNN-based classification model distinguished the soft and stiff MSCs by recognizing the bright peripheral and heterogeneous intracellular regions, respectively (Fig. 3h).

We used a CNN-based classification model to evaluate the intermediate stiffness of wt RAW264.7 cells. We correlated AFM-measured Young’s modulus at the single-cell level with stiffness ranges predicted by the CNN model. To quantify agreement, we fitted the data using a one-phase decay function (Fig. 3f) and calculated the APE between AFM-measured and model-predicted Young’s modulus values. Notably, the mean APE for both sets of values was found to be approximately 40% (Fig. 3g), while the coefficient of variation for AFM measurements on RAW264.7 was 15% (Fig. S4b), indicating reasonable consistency between experimental and predicted values. At the population level, we treated wt RAW264.7 cells with LPS and IL-4 to induce M1 and M2 macrophage phenotypes. We confirmed these phenotypes using iNOS (M1) and Arginase-1 (M2) via immunofluorescence (IF) and flow cytometry (FACS) (Fig. 3j, k). Using the CNN model and AFM, we evaluated the stiffness of different macrophage phenotypes. Consistent with AFM measurements, M1 macrophages exhibited higher stiffness than M0/M2 phenotypes, with no significant difference between M0 and M2 stiffness (Fig. 3i). Overall, this RAW264.7 stiffness classification model also possessed the predictive power to evaluate the intermediate stiffness of RAW264.7, indicating that evaluation of cell stiffness using cell type-specific stiffness classification models can be generalized to different cell types.

### Applications of MSC and macrophage stiffness classification models

**Fig. 4:**
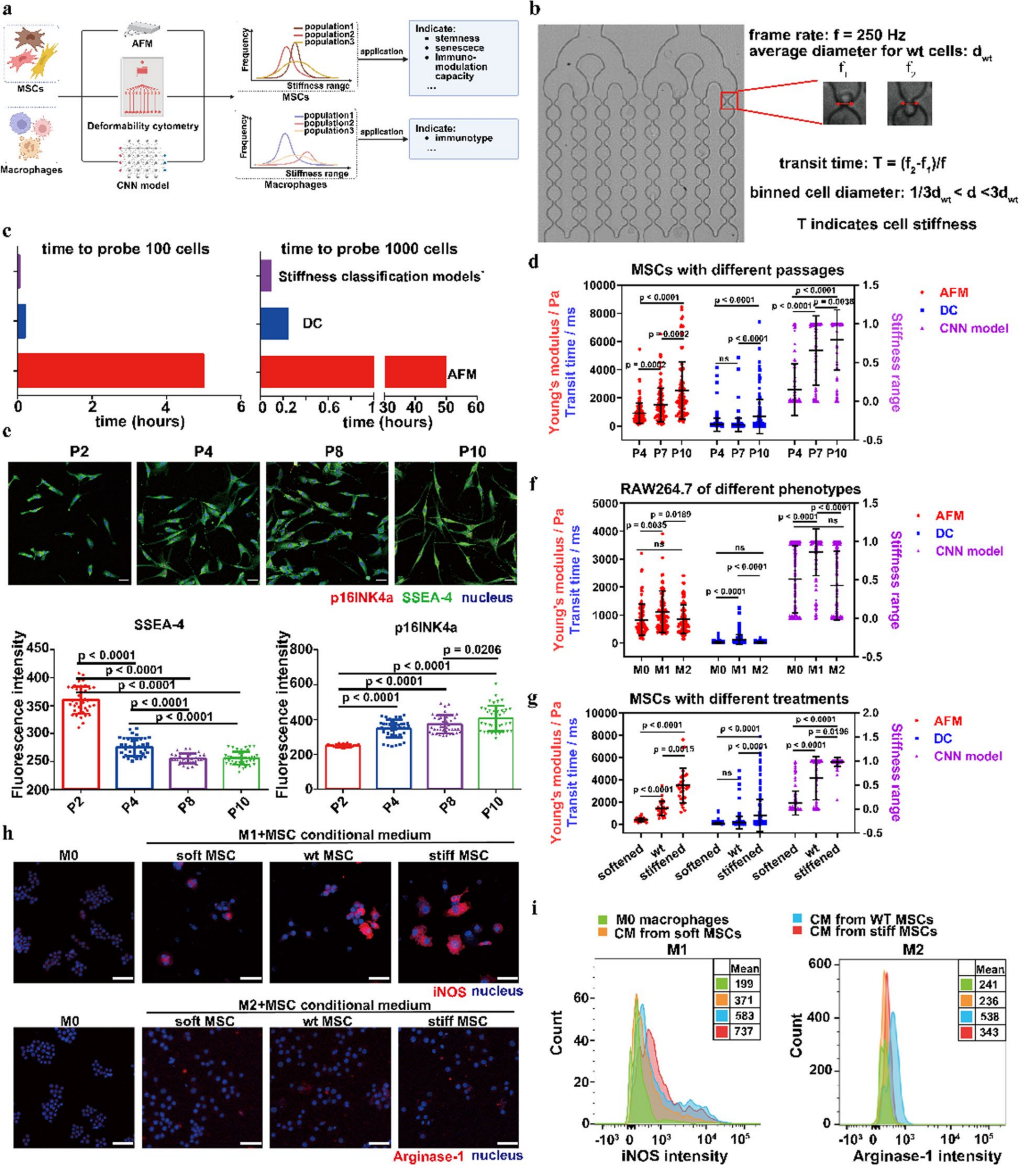
Applications of MSC and macrophage stiffness classification models. **a.** Schematic for the workflows. The stiffness classification models were applied to explore the correlations between cell stiffness and functions or phenotypes for MSCs and macrophages respectively. **b.** DC measurements for cell stiffness. The transit time in the first constriction region was chosen to reflect the stiffness of cells with binned diameters. **c.** Throughput comparison for AFM, DC, and stiffness classification models for characterization of the stiffness of 100 and 1000 cells. **d.** Stiffness evaluation for MSCs with different passages using AFM, DC, and MSC stiffness classification model. **e.** Characterizing MSC stemness (SSEA-4) and senescence (p16INK4a) using IF staining. **f.** Stiffness evaluation for macrophage with different phenotypes using AFM, DC, and RAW264.7 stiffness classification model. **g.** Stiffness evaluation for MSCs treated with 25 mM Bleb for 8 h and 0.15 mM H_2_O_2_ for 5 days using AFM, DC, and the MSC stiffness classification model. **h-i.** Characterizing phenotypes of M1 and M2 macrophages after treatment with CMs from MSC subpopulations for 48 h using IF and FACS. n ≥ 3 per group. Statistical analysis was performed using one-way ANOVA with Tukey’s test and the Kruskal–Wallis test. Results are presented as mean ± SD

Following the construction of our stiffness classification models, we conducted a comparison between the efficiency and accuracy of models with two commonly used techniques for mechanical phenotyping—DC and AFM—on both MSCs and macrophages at the population level (Fig. 4a). DC can evaluate cell stiffness with high throughput (Lange et al. 2015). In this work, we directly used the transit time for cells to pass through the first constriction region to represent the degree of cell stiffness by binning the cell diameters (Fig. 4b)(Mietke et al. 2015). While AFM is capable of measuring only tens of cells per hour, both DC and deep learning-based stiffness classification models have much higher throughput, surpassing thousands of cells per minute (Fig. 4c). We first evaluated MSC stiffness with different passages cultured in vitro using AFM, DC, and our MSC stiffness classification model. Overall, the findings from these three approaches consistently demonstrated a significant increase in the stiffness of MSCs with each passage. Specifically, MSCs in the 7th generation (P7) were significantly more rigid than MSCs in the 4th generation (P4) based on the MSC stiffness classification model, which is consistent with the results from AFM measurements. However, DC could not detect the stiffness difference between P4 and P7 MSCs (Fig. 4d). We hypothesized that the specific configuration of microfluidic-based DC (e.g., the width of constriction region and the flow velocity) could potentially hinder its ability to differentiate MSC subpopulations characterized with relatively low stiffness (Video S1, Fig. S5a). For instance, softer MSC subpopulations passed through the constriction regions too quickly for the camera to capture the difference in transit times.

Furthermore, we performed additional characterization of MSCs from different generations using SSEA-4 as a marker for stemness and p16INK4a as a marker for senescence. Our results demonstrated a progressive decrease in MSC stemness and an increase in senescence with each passage (Fig. 4e). Also, MSCs from different donors exhibited consistent changes in stiffness, stemness, and senescence (Fig. S6). As a result, the MSC stiffness classification model has the potential to be utilized for the characterization of cell stiffness with high throughput and sensitivity. This characterization for stiffness can, in turn, serve as an indication of cell stemness and senescence levels, thus facilitating MSC standardization and manufacturing processes. We then evaluated the stiffness of different phenotypes of macrophages using AFM, DC, and the RAW264.7 stiffness classification model respectively. The experimental setup of DC for RAW264.7 stiffness measurement was designed specifically for RAW264.7 cells (Video S2, Fig. S5b). Similarly, it was observed that M1 phenotypes of macrophages exhibited higher stiffness compared to M0 and M2 phenotypes of macrophages. On the other hand, M0 and M2 phenotypes of macrophages demonstrated similar stiffness levels (Fig. 4f). This result is consistent with pre-existing research (Patel et al. 2012). In summary, the RAW264.7 stiffness classification model has the potential to be employed for the characterization of cell stiffness, and subsequently serve as an indicator of the phenotypic and functional diversity of macrophages, in a high-throughput manner. By utilizing this model, researchers can gain insights into the relationship between macrophage stiffness and their specific phenotypes, thereby contributing to a better understanding of their diverse functions and behaviors.

Furthermore, we characterized the interactions between MSCs exhibiting different stiffness levels and different phenotypes of macrophages. This experimental approach could enable us to effectively evaluate the immunomodulatory capacity of MSCs. First, we employed Bleb and H_2_O_2_ for MSC stiffness modulation to generate soft and stiff subpopulations respectively. The stiffness of these MSC subpopulations was assessed using AFM, DC, and the MSC stiffness classification model. Similar to our previous findings, the results obtained from the MSC stiffness classification model were consistent with AFM measurements while DC could not detect the stiffness difference between wt and softened MSC subpopulations (Fig. 4g). Next, we obtained the conditioned mediums (CM) from different subpopulations of MSCs, ensuring that there were no significant differences in cell count (Fig. S7). These CMs were then used to treat induced M1 and M2 macrophages. To characterize the resulting phenotype transitions, we evaluated the expression of iNOS for the pro-inflammatory M1 phenotype and Arginase-1 for the pro-healing M2 phenotype after treatment with MSC-derived CMs (Fig. 4h, i). Notably, we observed that the treatment with CMs derived from stiff MSC subpopulations could promote the M1 phenotype of macrophages, while CMs derived from soft MSC subpopulations tended to suppress the M1 phenotype of macrophages. Interestingly, CMs derived from the untreated wt MSC subpopulations might have enhanced the M2 phenotype of macrophages in our study. Hence, our findings highlight that MSC stiffness correlates with their immunomodulatory capacity on macrophages. Furthermore, the MSC stiffness classification model can be utilized to efficiently characterize cell stiffness, enabling high-throughput and highly sensitive evaluation. This approach provides valuable insights into cell functions, including MSC stemness, senescence, and immunomodulatory capacity and holds promising potential for quality assessment of cell-based therapeutic products, enhancing our understanding of their functionality and therapeutic potential.

### Image-based evaluation of stiffness values across cell types using the deep learning-based regression model

**Fig. 5:**
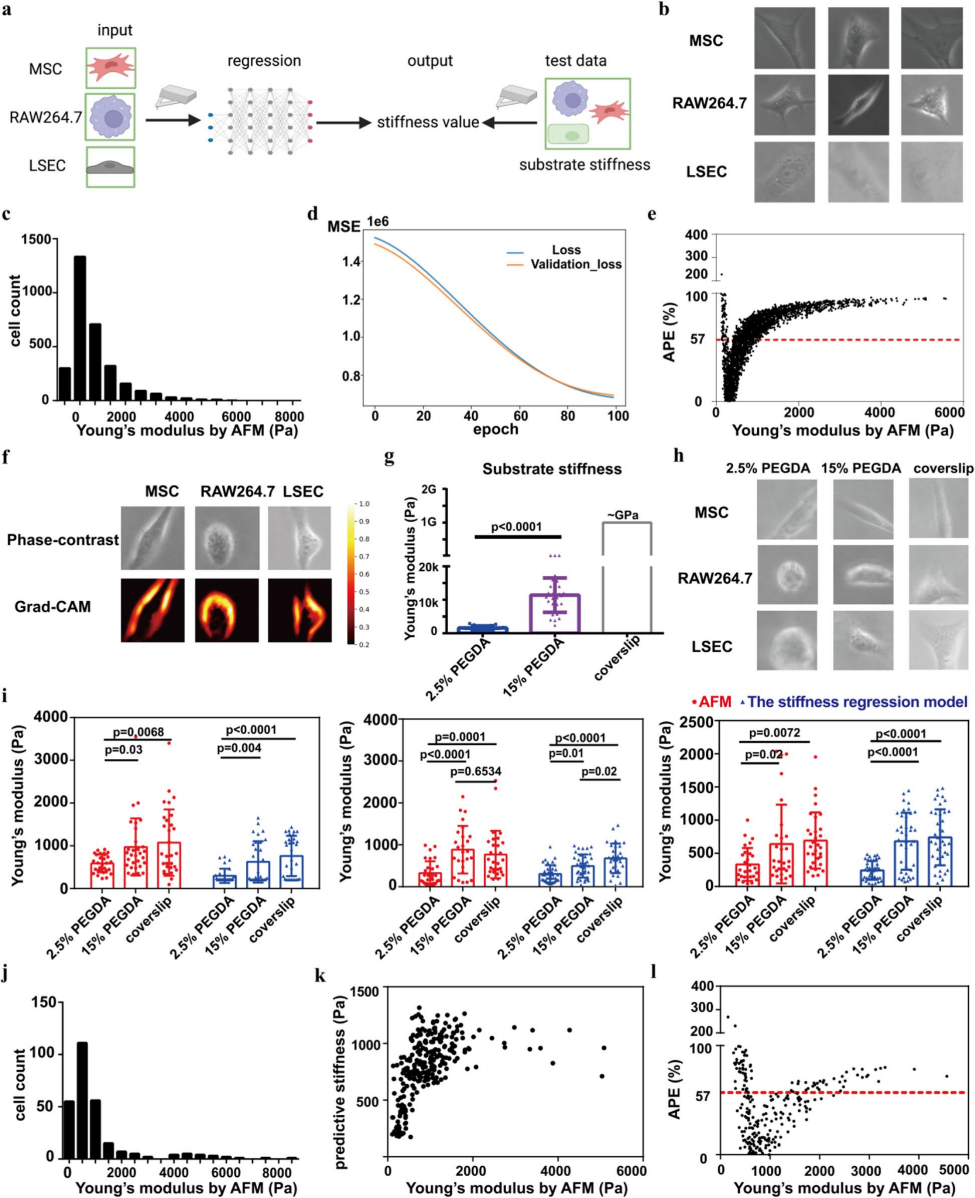
Image-based evaluation of stiffness values using the stiffness regression model. **a.** Schematic for 788 model establishment. The regression model was trained with single-cell images of MSCs, RAW264.7, and LSECs with Young’s modulus by AFM and tested with cells cocultured on substrates with varied stiffness. **b-c.** The training set including cell images and corresponding Young’s modulus. **d.** The learning curve using MSE as the loss function. **e.** The APEs between the true and predictive Young’s modulus for the training set. The red dot line indicates the mean. **f.** The modified Grad-CAM showed important regions that decreased the predictions while the predictive value was larger than the true value. **g.** Substrate stiffness measured with AFM. **h.** The test data including cropped single-cell images cocultured on substrates. **i.** Stiffness evaluation for cells cocultured on substrates with varied stiffness. **j.** Young’s modulus for the test data. **k.** Scatter plot for the true and predictive Young’s modulus for test data. **l.** The APEs between true and predictive Young’s modulus for the test data. The red dot line indicates the mean. Data are not statistically significant when p values are not shown; n ≥ 3 per group

We have developed deep learning-based classification models to evaluate cell stiffness, focusing on MSCs and macrophages. These models can assess cell stiffness with high throughput and sensitivity. Previous studies identified general image features indicating cell stiffness (Fig. S8), but we aimed to advance this by creating an image-based regression model to directly predict Young’s modulus across cell types using deep learning. However, dataset limitations posed a challenge. Due to the slow measurement rate of AFM, we conducted a proof-of-concept study with a limited dataset. We included LSECs along with MSCs and RAW264.7 cells for stiffness measurements. The regression model was trained and validated using single cells on substrates with varying stiffness (Fig. 5a). In total, we captured images of 1,149 MSCs, 1,237 RAW264.7 cells, and 939 LSECs, with their Young’s moduli measured by AFM (Fig. 5b, c). Despite the small sample size, we divided the data into training and validation sets (8:2 ratio) and tested the model in independent experiments.

For training the regression model, we employed MSE as the loss function and monitored the learning curve (Fig. 5d). The mean of the APEs for the regression model was 57%, which was higher than the performance of the CNN-based stiffness classification model after converting stiffness ranges to Young’s modulus (Fig. 5e). To understand the model’s decision-making, we applied a modified Grad-CAM algorithm to visualize the key regions influencing predictions (Kanda et al. 2020). Consistent with previous findings from stiffness classification models, the Grad-CAM revealed that the model reduced its output by focusing on bright peripheral regions when overestimating Young’s modulus (Fig. 5f). Additionally, it highlighted that the model increased its output by identifying heterogeneous intracellular regions, particularly in MSCs, when underestimating Young’s modulus (Fig. S9). This visualization provided insights into how the model adjusts its predictions based on cellular features.

Based on these observations, we hypothesized that the regression model might struggle to distinguish subtle stiffness differences between softer and stiffer RAW264.7 or LSECs. This difficulty likely stems from the limited dataset. To test the model, we used PEGDA hydrogels, the average Young’s modulus for 2.5% PEGDA was 1 kPa, while that for 15% PEGDA was 10 kPa (Fig. 5g). MSCs, RAW264.7 cells, and LSECs were cultured on these substrates, and single-cell images were captured (Fig. 5h). Their corresponding Young’s modulus were measured using AFM and predicted by stiffness regression model (Fig. 5j). The model’s predictions aligned with AFM results: P4 MSCs were stiffer on glass than on PEGDA, while RAW264.7 cells and LSECs were stiffer on 15% PEGDA than on glass (Fig. 5i). This shows the model can predict stiffness differences across these cell types and the mean of APEs.

We hypothesized that cell stiffness responses to substrate stiffness changes might be influenced by the intrinsic stiffness of specific cell types. We assessed late-passage (P9) MSCs on these substrates using AFM and the regression model. The results revealed that P9 MSCs were stiffer than P4 MSCs, and the model captured this change (Fig. S10). However, predicted Young’s modulus values were consistently lower than AFM measurements, likely due to the limited dataset. Notably, only a small subset of MSCs showed higher modulus values (Fig. 5c), which may have limited the model’s predictive power. The correlation between predicted and AFM-measured values was strong within 200–1,200 Pa but weaker at higher values (Fig. 5k). The test set’s mean APE was 57%, matching the overall dataset’s mean APE (Fig. 5l).

In summary, this deep learning-based regression model demonstrated some predictive capability in evaluating cell stiffness differences across various cell types. While the limited dataset size posed challenges in achieving higher accuracy, the model provided valuable insights into the relationship between cell stiffness and substrate properties. Future studies could benefit from expanding the dataset and refining the model architecture to enhance its predictive performance.

## Discussion

In this work, we proposed to evaluate cell stiffness based on cell images using deep learning. We first constructed an image-based stiffness classification model to evaluate the intermediate stiffness of MSCs. The robust performance of this stiffness classification model was also generalized for evaluating the stiffness of RAW264.7 cells, exemplifying its versatility and applicability to various cell types. We further applied MSC and RAW264.7 stiffness classification models for cell-type-specific mechanical phenotyping and exploration of the correlation between cell stiffness and functionality. Then we conducted a proof-of-concept study by training a deep learning-based stiffness regression model across cell types. The model utilized single-cell images of MSCs, RAW264.7 cells, and LSECs, which were labeled with AFM-measured Young’s modulus values. Despite being trained on limited datasets, the model demonstrated predictive capability when assessing cocultured cells on substrates with varying stiffness. Combining the performance of classification and regression models, we found that while regression models theoretically offer greater accuracy with large datasets, our study was constrained by the low throughput of AFM measurements, yielding only about 3,000 data points, which posed challenges for regression performance. In contrast, the classification model benefits from the ability to generate large datasets under controlled chemical and physical conditions, even when these datasets are relatively simple and discontinuous. Consequently, the classification model’s output, when converted via functional transformation, outperformed the regression model’s predictions (APE of classification model = 45%, APE of regression model = 57%). It is proved that the classification model can learn key information about stiffness changes in cell images. This highlights that with limited data measurement, training an evaluation model using abundant, simpler data and later fine-tuning it with precise datasets can significantly reduce model construction complexity.

There is still room for further improvement in these deep learning models. Firstly, the dataset sizes used in training the models were not sufficient to cover the entire range of stiffness values. As a result, the classification model performed better in identifying the softer subpopulations of MSCs and macrophages when compared to the stiffer subpopulations. Limitations in versatility of cell modulation methods also contributed to the challenges regarding performance of the stiffness classification models. Specifically, the Young’s modulus of soft cell subpopulations was mainly located within a narrow range, making them more homogenous in terms of stiffness. On the other hand, the stiffness of the stiffened subpopulations exhibited larger variations, accounting for higher predictive errors. Secondly, the stiffness regression model's predictive power was limited due to two factors: the small dataset from low-throughput AFM measurements and significant human error during these measurements. To improve predictive power, future work should focus on more consistent measurement techniques and larger datasets. What is more, we will need to further validate and improve the generalizability of the stiffness classification models by adding more operators, imaging modes, and cell culture conditions. The use of an enlarged dataset can be beneficial in establishing a regression model that could potentially provide more accurate stiffness predictions and broader applications.

After constructing and evaluating the deep learning model, we validated the functionality of MSC stiffness classification model, finding that cell stiffness indicates various MSC functions in vitro and may serve as a biophysical marker of MSC status within diverse populations. We also assessed primary mouse bone marrow-derived macrophages (BMDMs) and RAW264.7 cells using AFM and a pre-trained RAW264.7 stiffness classification model. The model’s results aligned with AFM measurements, indicating BMDMs are stiffer than RAW264.7 cells (Fig. S11). This suggests the pre-trained model might predict primary macrophage stiffness, a hypothesis warranting further validation with diverse macrophage batches. Notably, M2 macrophages showed no stiffness difference compared to M0 macrophages. This might stem from heterogeneity in IL-4-induced M2 macrophages, which could obscure stiffness differences between M0 and M2 subsets (Li et al. 2022; Wynn et al. 2013). In the future, we aim to generalize our model to more cell types and link cell stiffness to cellular functions to enhance its application. Recent studies have shown that cell stiffness is significantly related to various biological functions. For example, in cancer research, changes in cell stiffness have been found to influence tumor progression and response to therapy. One study demonstrated that stiffening cancer cells through cholesterol depletion can enhance the efficacy of adoptive T-cell immunotherapy, indicating that cell stiffness can modulate immune cell interactions (Lei et al. 2021). In the context of immune responses, T cell stiffness changes during the formation of the immunological synapse, with T cell lamellipodia becoming stiffer when forming synapses with antibody-coated surfaces, and both lamellipodia and cell body stiffness increasing post-synapse formation (Jung et al. 2021). Regarding aging, ROCK inhibition has been shown to reduce cell stiffness and size in tendon stem/progenitor cells, reversing age-related changes and highlighting its therapeutic potential (Chen et al. 2018). Although our study does not include additional experiments to validate the CNN-predicted hardness values, the existing research strongly supports the correlation between cell stiffness and biological outcomes. The consistency between CNN predictions and established biological findings suggests that these predictions can be a valuable tool for understanding cellular behavior and potential therapeutic applications.

To gain a better understanding of the decision-making process of the model, we used the Grad-CAM visualization, which revealed that both models classified cell stiffness based on bright peripheral (soft) and heterogeneous intracellular regions (stiff), aligning with the regression model (Fig. S9). Despite applying various chemical and physical treatments, the models focused on stiffness differences rather than regulatory stimuli effects. We hypothesized that universal features in phase-contrast imaging, particularly bright peripheral regions in soft cells due to height differences, could indicate stiffness. Soft cells, with larger heights and weaker adhesion, showed these bright regions, while stiff cells might reveal mechanical properties through specific intracellular regions. The results are in accordance with previous studies (Wilhelm et al. 2014; Young et al. 2020).

Since cells possess complex mechanical properties apart from stiffness, we also aim to explore the feasibility of image-based evaluation of other mechanical properties (e.g., cell viscosity or subcellular mechanics) using deep learning in the future work. For example, the bulk viscosity of MSCs has been shown to correlate with their differentiation potential (González-Cruz et al. 2012). At subcellular levels, the nuclear viscoelasticity of MSCs determines their osteogenic differentiation (Matsushita et al. 2021). AFM is able to measure subcellular mechanics, and several approaches to probe cell viscoelasticity have been proposed (Efremov et al. 2017). Meanwhile, we can use timelapse Z-stack cell images as input to enhance our model’s performance. Image-based stiffness evaluation models using deep learning not only enable mechanical phenotyping with high throughput but also provide new insight into the underlying mechanism of the basis for single-cell mechanics.

In summary, we validated the feasibility of bright-field image-based cell stiffness evaluation using deep learning-based classification and regression models. These models enable high-throughput, in situ, and non-invasive evaluation of single-cell mechanics, facilitating the integration of stiffness evaluation as a predictive tool in various biological and biomedical applications. These models also provide new perspectives for fundamental cell biology research and clinical applications for therapeutic cell products.

## Methods

### Cell culture

Mesenchymal stem cells (MSCs) were isolated from human adipose, bone marrow, and umbilical cord. MSCs were cultured in the mesenchymal stem cell growth medium (Cat# M005, BioWit Technologies). Mouse macrophage cell line (RAW264.7) were cultured in mediums composed of high glucose Dulbecco’s modified Eagle medium (4.5 g/L glucose, Wisent, Canada) supplemented with 10% FBS (Cat# 087–150, Wisent) and 1% penicillin–streptomycin (Cat# 450–201-CL, Wisent). MSCs and RAW264.7 were cultured in a humidified 5% CO_2_ incubator (Thermo Fisher) at 37 °C. Cell culture work for individual cell types was performed by two operators separately.

### Microscopic imaging

Phase-contrast images of cells were acquired using phase contrast objective lenses with the included Ph1 phase annulus on an inverted Nikon Eclipse Ti-S microscope. The motorized live-cell imaging system enabled high-speed and fully automated cell imaging with 10X objectives for MSCs and 20X objectives for RAW264.7. For the training datasets in cell stiffness classification models, we acquired the phase-contrast images of soft and stiff MSCs and RAW264.7 cells. Images of soft, wt, and stiff cells were used as the test datasets. All images were acquired from over three independent experiments and saved as 1392*1040 px RGB images in Tiff format. This part was finished by two operators independently.

### Atomic Force Microscopy (AFM) for Young’s modulus measurements and cell imaging

Single-cell imaging and Young’s modulus measurements were conducted using an AFM module (Nanowizard, JPK Instruments) mounted on an inverted fluorescence microscope (Zeiss Observer A1). Silicon nitride cantilevers (DNP-10, Bruker) with a nominal spring constant of 0.06 N/m were used. Cells were seeded at low density on gelatin-coated 25 mm coverslips overnight. AFM cantilevers were calibrated using the thermal vibration method, and the spring constant was determined by fitting the thermal spectrum with a Lorentzian function. Cells were imaged using a 20X phase contrast objective (Nikon) with Ph1 phase annulus. Young’s modulus was calculated by fitting force-versus-indentation curves to the Hertz model using JPKSPM Data Processing software, with three measurements averaged per cell. All experiments used consistent parameters, and each group included at least 30 sample points.

### Deformability cytometry (DC)

The DC setup was built following a published report (Lange 2015). The microfluidic device setup was fabricated using soft lithography on a 4-inch silicon wafer, with a geometry designed to properly deform cells. The constriction widths were 5 μm for RAW264.7 cells and 6.5 μm for MSCs. PDMS devices were prepared from a 10:1 base-to-curing agent mixture, cured overnight at 65 °C, and bonded to coverslips using plasma treatment for 3 min. The microfluidic chips were mounted on an inverted microscope (Zeiss) equipped with a 20X objective and a high-speed camera. Cell samples were digested, resuspended in 0.01% Pluronic F-127, and flowed through the microfluidic chamber at rates of 20 μL/min for MSCs and 10 μL/min for RAW264.7 cells using a programmable syringe pump. Cell displacement and deformation were recorded at 250 frames/s in a 128 × 256 pixel region of interest. Transit time through the first constriction region, which depends on cell stiffness and size, was extracted as an indicator of single-cell stiffness by analyzing cells of similar diameters.

### Modulation of cell Young’s modulus

MSCs with passage numbers lower than 6 were chosen for the experiments. We decreased cell Young’s modulus using two cytoskeletal inhibitors: Cyto.D (Cat# S818402, Selleck) and Bleb (Cat# E124901, Selleck). We increased cell Young’s modulus using two stressors: glucose and H_2_O_2_. For the CNN training, MSCs were primed with 5 µM Cyto.D for 0.5 h or 25 µM Bleb for 2 h to obtain homogeneous soft MSC subpopulations. MSCs were cultured with 25 mM glucose or 0.15 mM H_2_O_2_ for 5 days to obtain stiff MSC subpopulations. Modulations of RAW264.7 cells were achieved with Cyto.D and H_2_O_2_. RAW264.7 cells were primed with 10 µM Cyto.D for 2 h to obtain soft RAW264.7 cells and treated with 0.5 mM H_2_O_2_ to obtain stiff RAW264.7 cells. Specifically, soft MSCs were modulated using 25 µM Bleb for 8 h to obtain their conditional medium. It significantly decreased MSC Young’s modulus similar to 25 µM Bleb treatment for 2 h.

### Immunofluorescence (IF) staining

Cell samples seeded in plates were fixed with 4% PFA for 15 min at room temperature. Then, cells were permeabilized with PBS containing 0.5% Triton X-100 (Sigma) for 10 min at 4 °C and blocked with PBS containing 1% BSA (Wisent) at room temperature for 1 h. Next, cells were incubated with primary antibodies(Abcam Cat# ab81278, Abcam Cat# ab16287, Cell Signaling Technology Cat# 13,120, Cell Signaling Technology Cat# 93,668) diluted in blocking solutions overnight at 4 °C. After extensive washes, cells were incubated with corresponding secondary antibodies (Cat# E032420, EarthOx) diluted in PBS for 2 h at room temperature, protected from light. Finally, cell nuclei were stained using Hoechst 33,342 (Sigma, 1:2000) diluted in PBS for 20 min at room temperature. Cell samples were exposed to 100 nM rhodamine-conjugated phalloidin (Cat# PHDR1, Cytoskeleton) for 30 min at room temperature for F-actin staining. Fluorescence imaging was performed using a Nikon Eclipse Ti-S microscope and ImageJ Fiji was used for subsequent image analysis.

### Flow cytometry (FACS) analysis

Cells cultured in plates were digested with trypsin to obtain suspended cell samples placed in 1.5 mL conical centrifuge tubes. Then cells were treated with the following reagents: 4% PFA for 15 min at room temperature; PBS containing 0.5% Triton X-100 for 10 min at 4 °C; PBS containing 1% BSA at room temperature for 1 h; blocking solution containing primary antibodies at room temperature for 1 h; PBS containing secondary antibodies at room temperature for 1 h. Cells were washed by centrifuging at 1000 rpm for 5 min and resuspending them with PBS during this process. Finally, the remaining immune-stained cells (> 10^4^) were analyzed using Flow cytometer LSRFortessa SORP (BD).

### GPU server and analysis environment

Our networks were trained with the Keras framework on NVIDIA GTX 1080Ti GPUs with CUDA 8.0, cuDNN 8.0, Anaconda 3 4.4.0, Python 3.7, Tensorflow 1.4.0, and Keras 2.1.2.

### Image data preprocessing

To generate single-cell images for training, we segmented cell images semi-automatically. Parts of single-cell images were segmented manually while part of images was cropped by the OpenCV-based script. Specifically, we remove the low-quality images without cells or with more than single cells. This work was performed by two operators independently. While the networks required uniform images as input, we resized each image to 50*50 px and divided each resized image pixel value by 255, subtract 0.5, and multiple by 2 to squash the value domain to −1 ~ 1. Also, we augmented the datasets with rotation, flip, and Gaussian blurring, to augment the datasets and avoid overfitting.

### Model training

The stiffness classification model in this work was based on the CNN neural network, consisting of the convolution layers, pooling layers, and fully-connected layers. RLU activation function was selected in the previous layers and softmax was selected as the activation function in the final layer. Adam algorithm as the optimizer and categorical_crossentropy as the loss function were chosen for model training with learning rates 1e-5. When applying the stiffness classification model to intermediate cell stiffness evaluation, we obtained the output probability that single MSCs belonged to the stiff class by the softmax function in the last layer as the predictive stiffness ranges (Fig. S12). In this work, we selected classical classification models (AlexNet, Inception V3, VGG16, and ResNet50) to perform stiffness classification tasks for MSCs. Inception V3, VGG16 and ResNet50 were pre-trained in ImageNet.

The stiffness regression model was based on the CNN neural network, consisting of one 3 × 3 convolution layer, two 2 × 2 pooling layers, and two fully-connected layers. RLU activation function was selected in the previous layers and linear activation was selected as the activation function in the final layer. Adam algorithm as the optimizer and MSE as the loss function were chosen for model training with learning rates 1e-5 (Fig. S13).

### Model evaluation

For the stiffness classification models of MSC and RAW264.7 macrophages, Indexes including accuracy, precision, recall and F1_score, confusion matrix, Receiver Operating Characteristic Curve and corresponding Area Under Curve were selected to evaluate the classification efficiency and the generalization ability of the models. For the cell stiffness classification model, the APEs between the predicted value and the measured value using AFM were calculated to evaluate the model performance in evaluating cell stiffness at the single cell level.

### Grad-CAM visualization for the CNN-based models

Referring to the published work (Selvaraju et al. 2020), we defined the parameter $d$ as follows:

$$d=\frac{1}{abs\left(x-{x}^{\prime}\right)}$$

$x$ was the model output and ${x}^{\prime}$ was the stiffness value measured with AFM. Then the gradients changes:

$${\alpha }_{k}^{c}=\frac{1}{z}\sum_{i}\sum_{j}\frac{\partial d}{\partial {A}_{ij}^{k}}$$

The channel k in feature layer A has the size Z (weight $\times$ height). The gradient for d in feature map A is:

$$\frac{\partial d}{\partial {A}_{ij}^{k}}=\frac{\partial d}{\partial x}\frac{\partial x}{\partial {A}_{ij}^{k}}$$

When the predictive value is larger than the true value,

$$\frac{\partial d}{\partial x}=-\frac{1}{{\left(x-{x}^{\prime}\right)}^{2}}<0$$

Grad-CAM visualizes the important regions to decrease the output. When the predictive value is smaller than the true value, Grad-CAM visualizes the important regions to increase the output.

### Fabrication of PEGDA hydrogels with controllable stiffness

To prepare PEGDA hydrogels with controllable stiffness, we first modified 25 mm coverslips by cleaning them with 10% NaOH at 90 °C for 20 min, followed by three washes with 75% ethanol. The coverslips were then treated with 10% TMSPMA for 30 min at room temperature to enhance hydrophilicity and rinsed with alcohol. Next, we prepared the PEG hydrogel solution by mixing 10% w/v APS, 10% v/v TMSPMA, and 20% w/v Am on ice, and added PEGDA to achieve the desired stiffness gradient. A 150μL aliquot of the mixture was placed on the center of each coverslip, and two horizontal PMMA plates were used to spread the solution evenly without bubbles. The coverslips were cured at 37 °C for 2 h. Finally, the coverslips were sterilized by immersion in 75% ethanol and exposure to UV light for 4 h. To promote cell adhesion, the surface of the coverslips was modified with a 0.1% gelatin solution at 4 °C overnight.

### Quantification and statistical analysis

Statistical analysis was performed using GraphPad Prism 6 software in the work. Multiple comparison between the groups was performed using Tukey’s multiple comparisons test in one-way ANOVA or Kruskal–Wallis test according to whether the sample population followed normal distribution or not. For experiments comparing two groups, we performed a two-tailed paired Student’s t-test or Mann–Whitney U-test according to whether the sample population followed normal distribution or not. The Gaussian function was used to fit the distribution of Young's modulus of cell subpopulations. Data were obtained from at least 3 individual experiments and expressed as means ± SD. Differences were represented by the p-value. The number of cell samples in each experimental group was above 30.

## Supplementary Information

Supplementary Material 1. Fig. S1. Diverse approaches used to modulate MSC stiffness. Fig. S2. The workflow for image data preprocessing. Fig. S3. The learning curves for classical classification model using deep learning. Fig. S4. The CVs of AFM measurements for (a) MSC and (b) RAW264.7. Fig. S5. The experimental setup of DC measurements of the stiffness of (a) MSC and (b) RAW264.7. Fig. S6. The stiffness and functions of MSCs from different donors. Fig. S7. The changes of cell number with time for MSC subpopulations. Fig. S8. The predictive power of RAW264.7 stiffness classification model on MSCs. Fig. S9. Modified Grad-CAM visualization for the important regions that the regression model used for the prediction of MSC stiffness. Fig. S10. Stiffness evaluation for MSC P9 cocultured with RAW264.7 and LSECs on substrates with different stiffness using AFM and the stiffness regression model. Fig. S11. Stiffness evaluation for RAW264.7 cell lines and BMDMs using AFM and the RAW264.7 stiffness classification model. Fig. S12. The structure of stiffness classification model. Fig. S13. The structure of stiffness regression model.

Supplementary Material 2. Movie S1. DC measurements for MSC stiffness

Supplementary Material 3. Movie S2. DC measurements for RAW264.7 stiffness.

## Data Availability

All data are available from the corresponding author upon request. Single-cell images of MSCs and macrophages used in this study have been deposited in the Figshare database and the DOI is https://doi.org/10.6084/m9.figshare.24502771.v1. Codes for CNN training in this study have been deposited in a GitHub repository, and the DOI is https://github.com/DuLabcoder/WZZ.

## Abbreviations

AFM: Atomic Force Microscope
Am: Acrylamide
APE: Absolute Percentage Error
AUC: Area Under Curve
BMDM: Bone Marrow-Derived Macrophage
CV: Coefficient Of Variation
CNN: Convolutional Neural Network
DMSO: Dimethyl Sulfoxide
FBS: Fetal Bovine Serum
Grad-CAM: Gradient-Weighted Class Activation Mapping
LSEC: Liver Sinusoidal Endothelial Cell
MSE: Mean Square Error
MAPE: Mean Absolute Percentage Error
MSC: Mesenchymal Stem Cell
PBS: Phosphate Buffered Saline
PEGDA: Poly (Ethylene Glycol) Diacrylate
PDMS: Polydimethylsiloxane
PFA: Paraformaldehyde
PMMA: Polymethyl Methacrylate
ResNet: Residual Neural Network
ROC: Receiver Operator Characteristic
TMSPMA: 3-(Trimethoxysilyl)Propyl Methacrylate
