# Correction: Image-based evaluation of single-cell mechanics using deep learning

**DOI:** 10.1186/s13619-025-00251-z

**Published:** 2025-07-09

**Authors:** Zhaozhao Wu, Yiting Feng, Ran Bi, Zhiqiang Liu, Yudi Niu, Yuhong Jin, Wenjing Li, Huijun Chen, Yan Shi, Yanan Du

**Affiliations:** 1 School of Biomedical Engineering, Tsinghua University, Beijing, 100084 China; 2 Tsinghua-Peking Center for Life Sciences, Tsinghua University, Beijing, 100084 China; 3 School of Basic Medical Sciences, Tsinghua University, Beijing, 100084 China

**Correction: Cell Regeneration 14, 21 (2025)**

**https://doi.org/10.1186/s13619-025-00239-9**

Following publication of the original article (Wu et al. 2025), the authors identified errors in Fig. 4e and Fig. 4h that occurred during the figure assembly process. Specifically, the immunofluorescent images in these panels were inadvertently misplaced and duplicated. For instance, in Fig. 4e, the images for P2 and P8 were erroneously duplicated. In Fig. 4h, the images for soft MSC and stiff MSC in the M1 + MSC conditioned medium group, as well as the images for wt MSC in the M1 + MSC conditioned medium and M0 in the M2 + MSC conditioned medium, were also incorrectly duplicated. Additionally, the images for soft MSC and wt MSC in the M2 + MSC conditioned medium group were mistakenly replaced with images originally belonging to P8 in Fig. 4e.

The inadvertent duplication and misplacement of images in Fig. 4e and Fig. 4h were caused by an issue with Adobe Illustrator and were not the result of intentional manipulation. During final proofing while formatting images, Illustrator automatically replaced missing linked files with incorrect images without alerting the user. We confirm that this was a technical error during figure processing, with no scientific misconduct involved.

To address this issue, corrections have been made to Fig. 4e and Fig. 4h in this revision. The corrected figures now accurately reflect the intended experimental data. Importantly, these corrections are in alignment with the original versions of the article and do not affect the analyses, interpretations, or overall conclusions presented.

The incorrect Fig. 4 is:

**Fig. 4:**
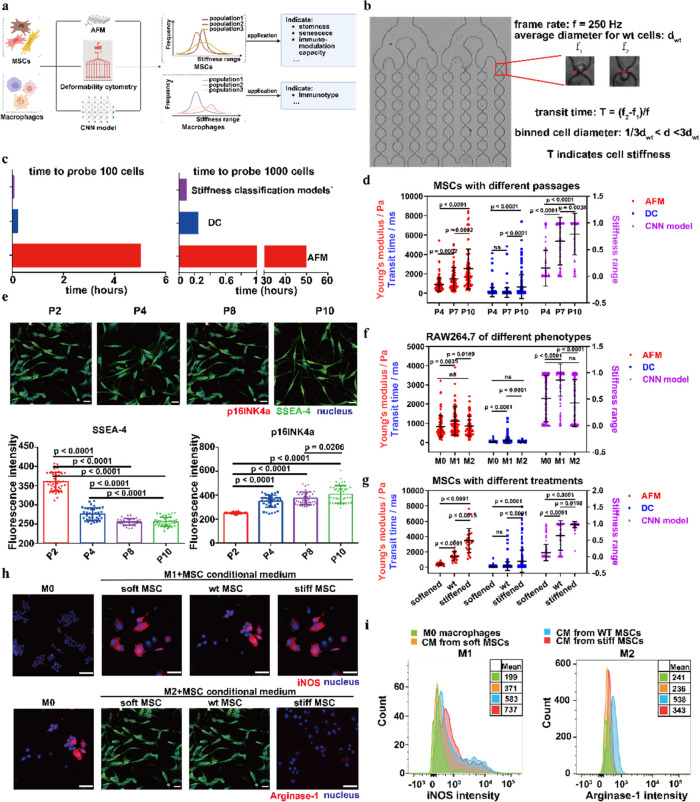
Applications of MSC and macrophage stiffness classification models. **a** Schematic for the workflows. The stiffness classification models were applied to explore the correlations between cell stiffness and functions or phenotypes for MSCs and macrophages respectively. **b** DC measurements for cell stiffness. The transit time in the first constriction region was chosen to reflect the stiffness of cells with binned diameters. **c** Throughput comparison for AFM, DC, and stiffness classification models for characterization of the stiffness of 100 and 1000 cells. **d **Stiffness evaluation for MSCs with different passages using AFM, DC, and MSC stiffness classification model. **e** Characterizing MSC stemness (SSEA-4) and senescence (p16INK4a) using IF staining. **f** Stiffness evaluation for macrophage with different phenotypes using AFM, DC, and RAW264.7 stiffness classification model. **g** Stiffness evaluation for MSCs treated with 25 mM Bleb for 8 h and 0.15 mM H2O2 for 5 days using AFM, DC, and the MSC stiffness classification model. **h**-**i** Characterizing phenotypes of M1 and M2 macrophages after treatment with CMs from MSC subpopulations for 48 h using IF and FACS. *n* ≥ 3 per group. Statistical analysis was performed using one-way ANOVA with Turkey’s test and the Kruskal–Wallis test. Results are presented as mean ± SD

The correct Fig. 4 is:

**Fig. 4:**
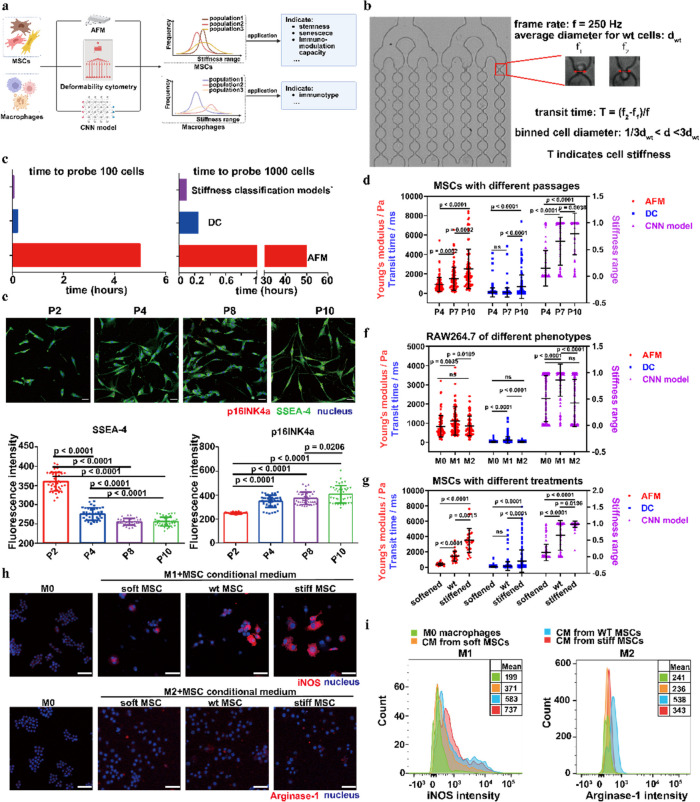
Applications of MSC and macrophage stiffness classification models. **a** Schematic for the workflows. The stiffness classification models were applied to explore the correlations between cell stiffness and functions or phenotypes for MSCs and macrophages respectively. **b** DC measurements for cell stiffness. The transit time in the first constriction region was chosen to reflect the stiffness of cells with binned diameters. **c** Throughput comparison for AFM, DC, and stiffness classification models for characterization of the stiffness of 100 and 1000 cells. **d** Stiffness evaluation for MSCs with different passages using AFM, DC, and MSC stiffness classification model. **e** Characterizing MSC stemness (SSEA-4) and senescence (p16INK4a) using IF staining. **f** Stiffness evaluation for macrophage with different phenotypes using AFM, DC, and RAW264.7 stiffness classification model. **g** Stiffness evaluation for MSCs treated with 25 mM Bleb for 8 h and 0.15 mM H2O2 for 5 days using AFM, DC, and the MSC stiffness classification model. **h**-**i** Characterizing phenotypes of M1 and M2 macrophages after treatment with CMs from MSC subpopulations for 48 h using IF and FACS. *n* ≥ 3 per group. Statistical analysis was performed using one-way ANOVA with Tukey’s test and the Kruskal–Wallis test. Results are presented as mean ± SD

The original article (Wu et al. 2025) has been corrected.
