# Supplementary material for: Image-based evaluation of single-cell mechanics using deep learning
Source: Cell Regen. 2025 Jun 5;14:21. doi: 10.1186/s13619-025-00239-9 (PMC12137843; doi:10.1186/s13619-025-00239-9)
Supplement: Supplementary file 1 — Supplementary Material 1. Fig. S1. Diverse approaches used to modulate MSC stiffness. Fig. S2. The workflow for image data preprocessing. Fig. S3. The learning curves for classical classification model using deep learning. Fig. S4. The CVs of AFM measurements for (a) MSC and (b) RAW264.7. Fig. S5. The experimental setup of DC measurements of the stiffness of (a) MSC and (b) RAW264.7. Fig. S6. The stiffness and functions of MSCs from different donors. Fig. S7. The changes of cell number with time for MSC subpopulations. Fig. S8. The predictive power of RAW264.7 stiffness classification model on MSCs. Fig. S9. Modified Grad-CAM visualization for the important regions that the regression model used for the prediction of MSC stiffness. Fig. S10. Stiffness evaluation for MSC P9 cocultured with RAW264.7 and LSECs on substrates with different stiffness using AFM and the stiffness regression model. Fig. S11. Stiffness evaluation for RAW264.7 cell lines and BMDMs using AFM and the RAW264.7 stiffness classification model. Fig. S12. The structure of stiffness classification model. Fig. S13. The structure of stiffness regression model. [file 13619_2025_239_MOESM1_ESM.docx]

**Supplementary Information**


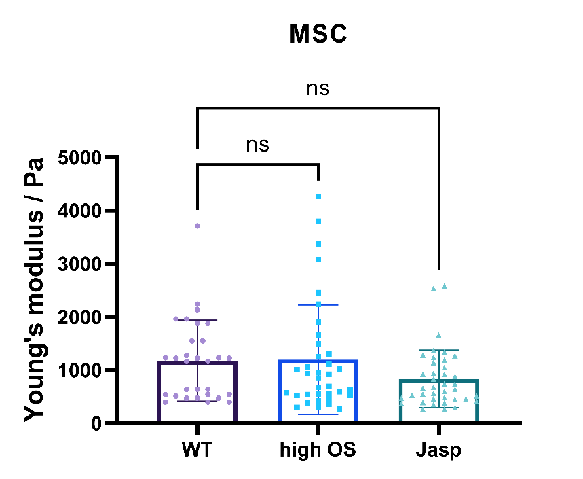


Fig. S1. Diverse approaches used to modulate MSC stiffness. MSCs were treated with high OS (5% PEG400) for 0.5 h and 0.5 𝜇M Jasp for 2 h, respectively.


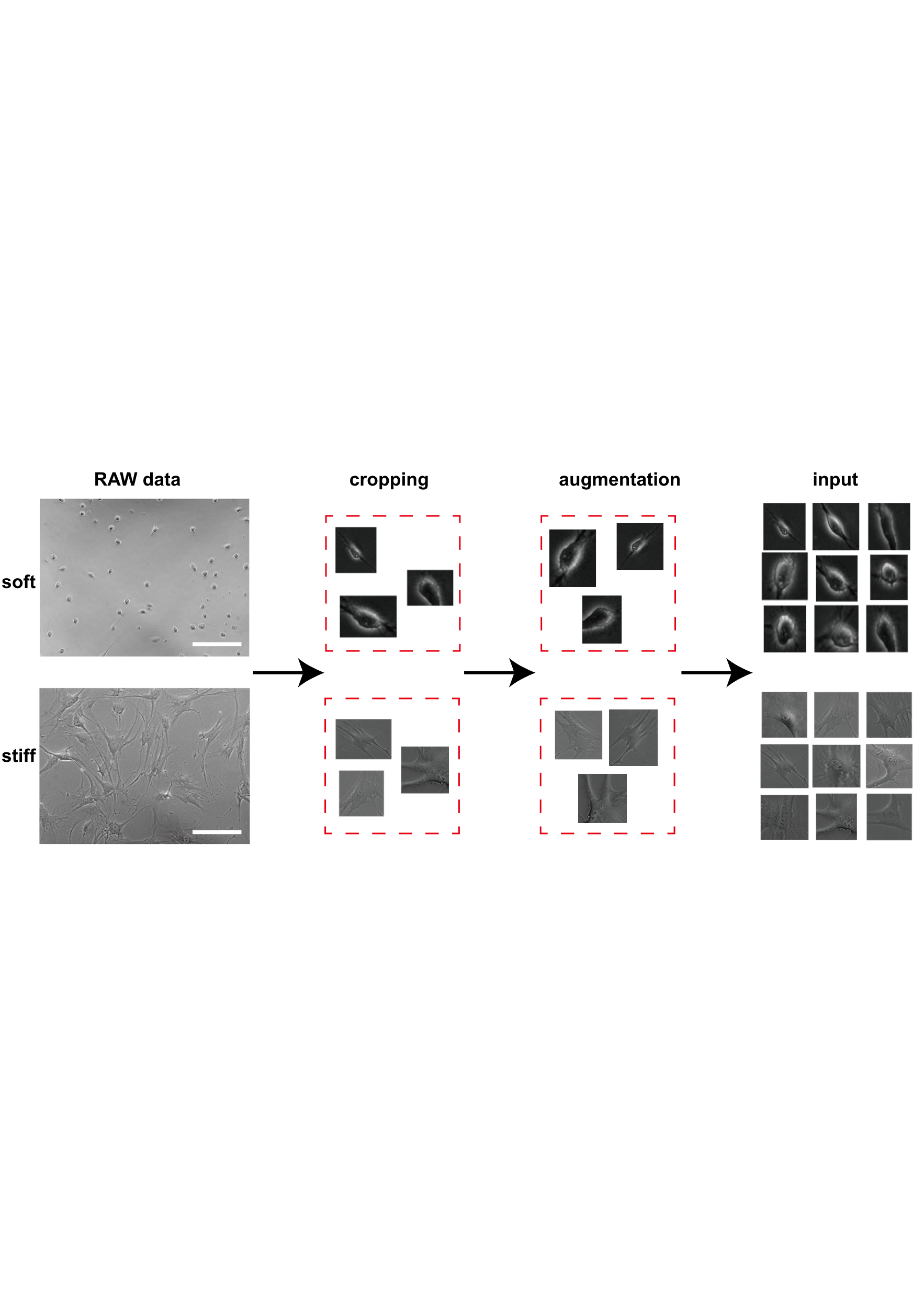


Fig. S2. The workflow for image data preprocessing. Single-cell images were cropped from populated subpopulations. Data augmentation was performed using ration, flip, and blurring. Then, images were resized to 50 * 50 px and normalized to -1 ~ 1 with (pixel/255 - 0.5) * 2.


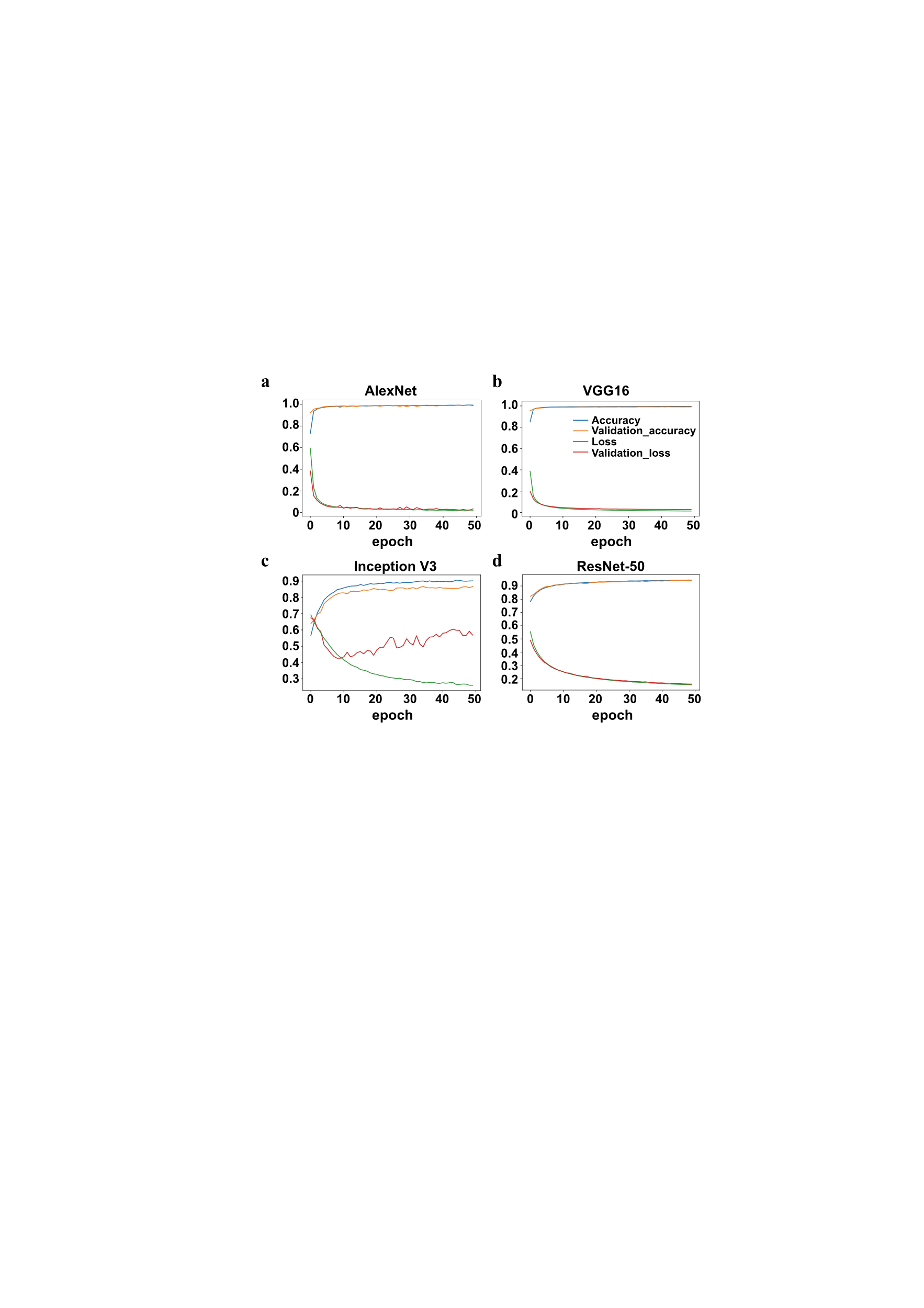


Fig. S3. The learning curves for classical classification model using deep learning. We trained the MSC stiffness classification models using (a) AlexNet, (b) VGG16, (c) Inception V3, and (d) ResNet-50, respectively.


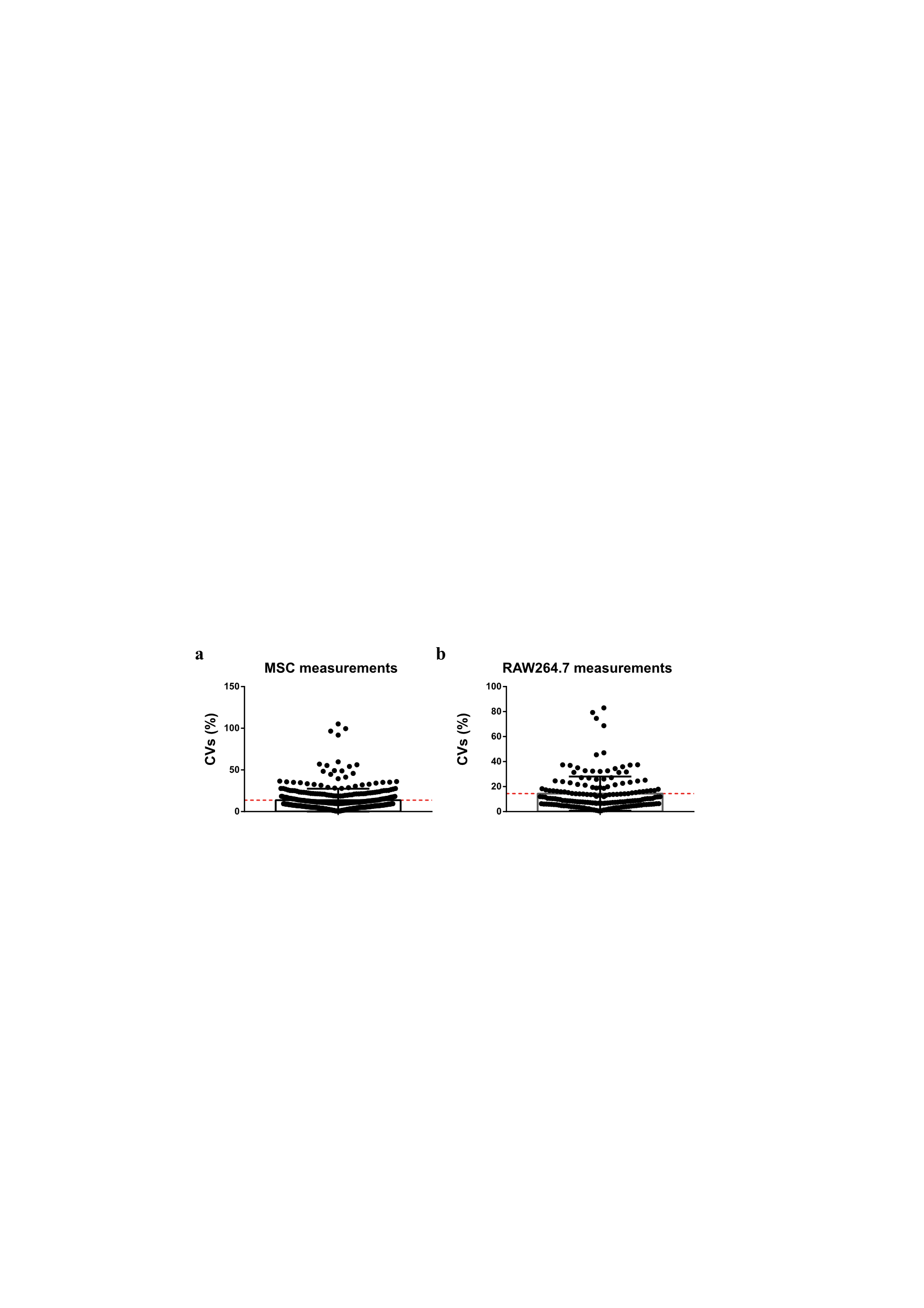


Fig. S4. The CVs of AFM measurements for (a) MSC and (b) RAW264.7. Each point represented a single cell and the red line indicated the mean of all CVs.


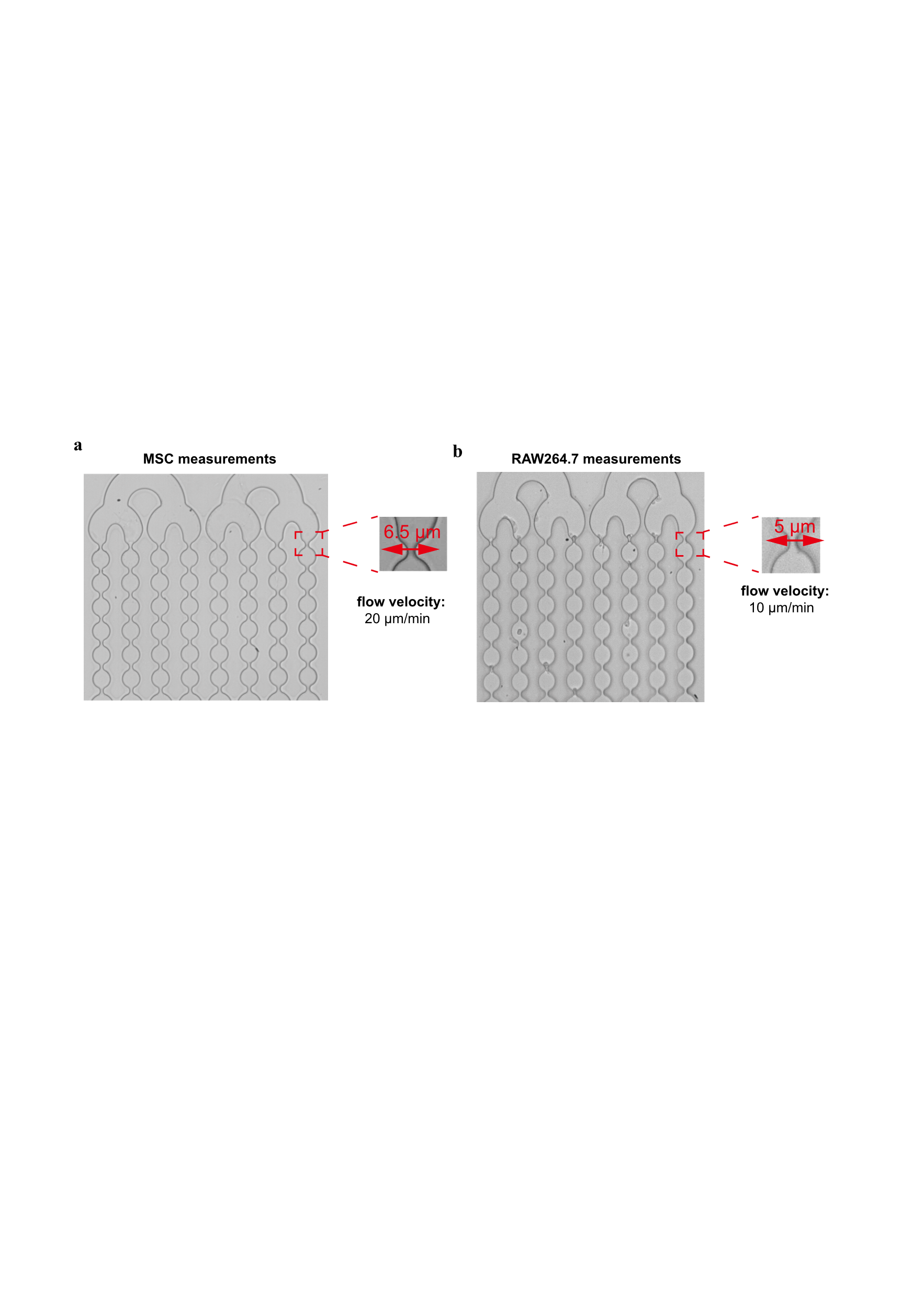


Fig. S5. The experimental setup of DC measurements of the stiffness of (a) MSC and (b) RAW264.7. The width of constriction regions and flow velocity were chosen mainly based on the difference in cell sizes.


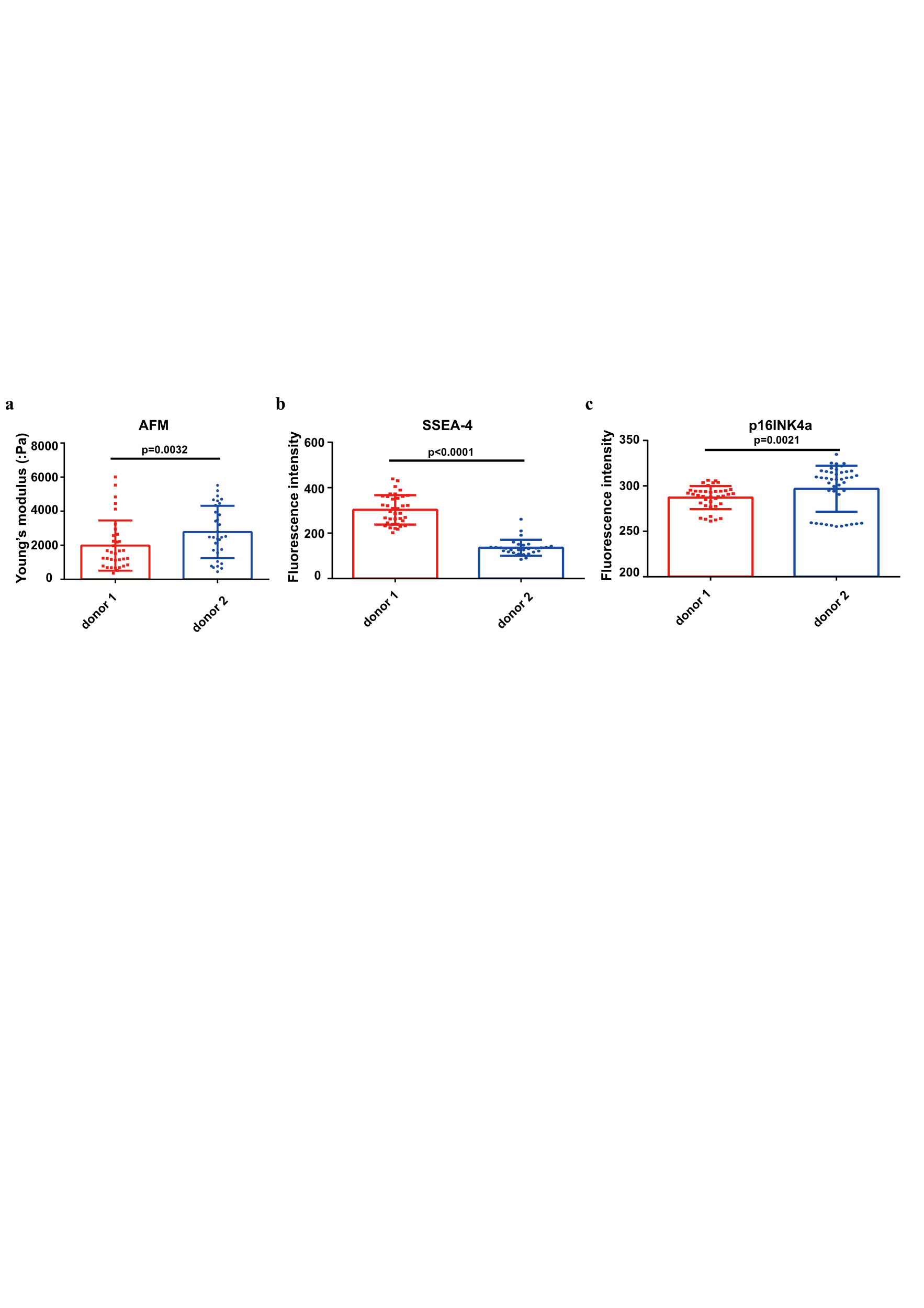


Fig. S6. The stiffness and functions of MSCs from different donors. (a) The difference in MSC Young’s modulus measured with AFM, (b) The difference in SSEA-4 fluorescence intensity, (c) The difference in p16INK4a fluorescence intensity.


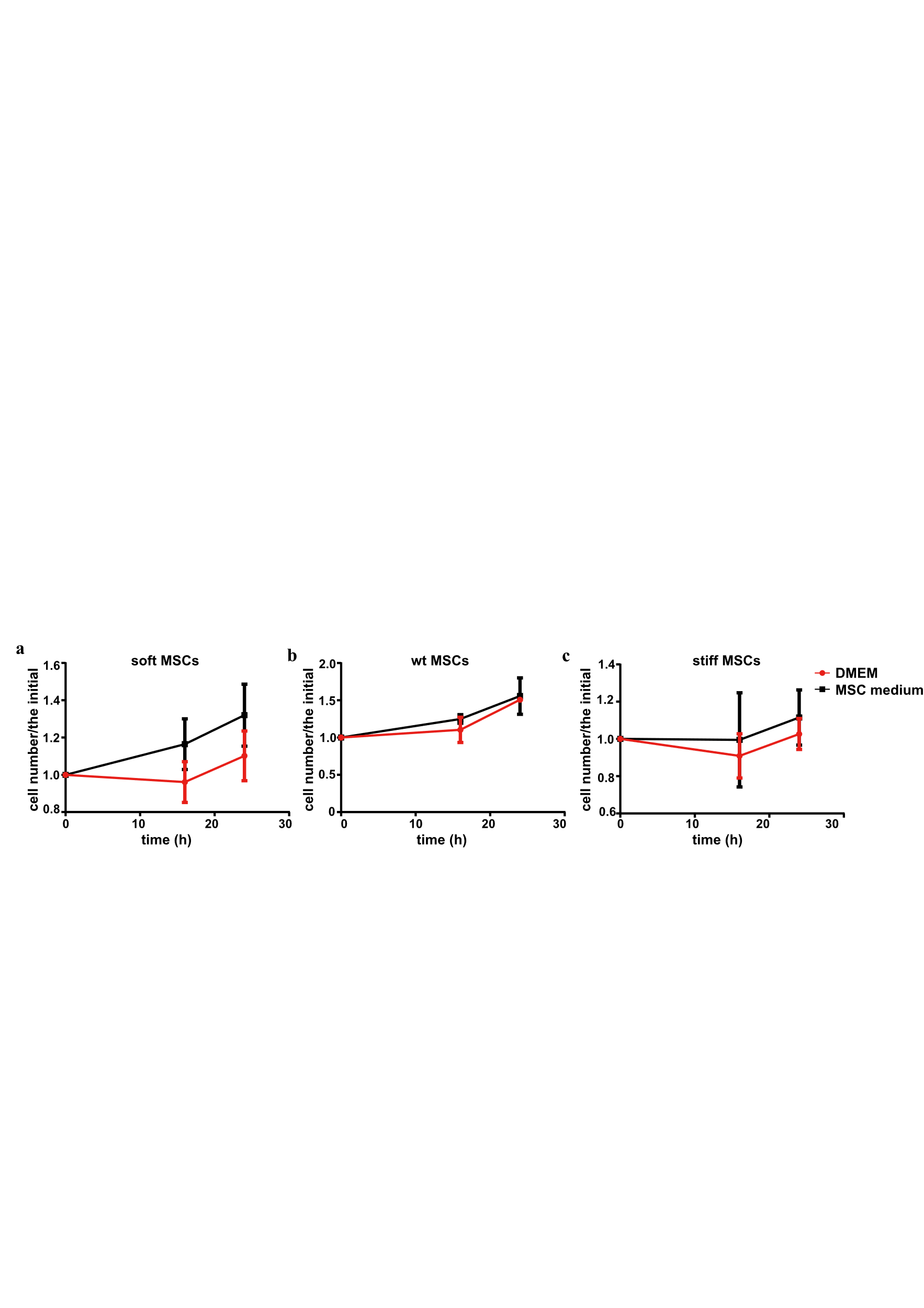


Fig. S7. The changes of cell number with time for MSC subpopulations. Changes in the ratio of (a) soft MSCs, (b) wt MSCs, and (c) wt stiff MSCs to the initial number (2*10^5^ after 2 h seeding) with time.


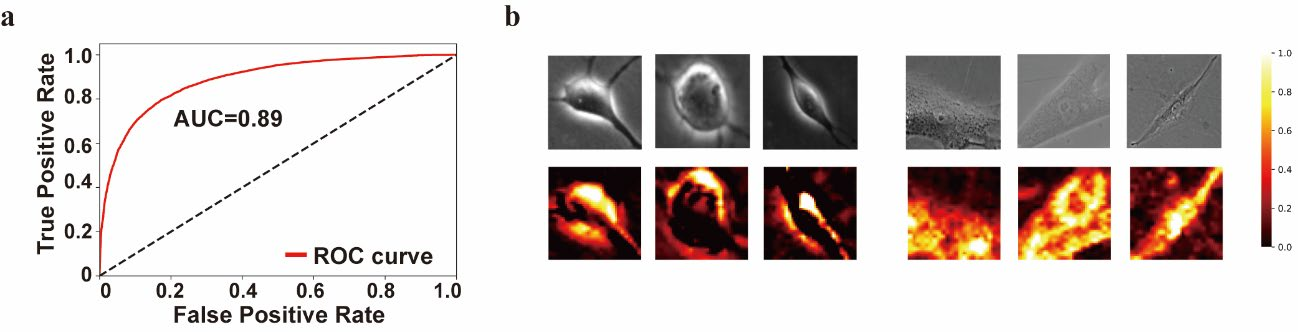


Fig. S8. The predictive power of RAW264.7 stiffness classification model on MSCs. (a) the ROC curve and (b) Grad-CAM visualization for soft and stiff MSCs.


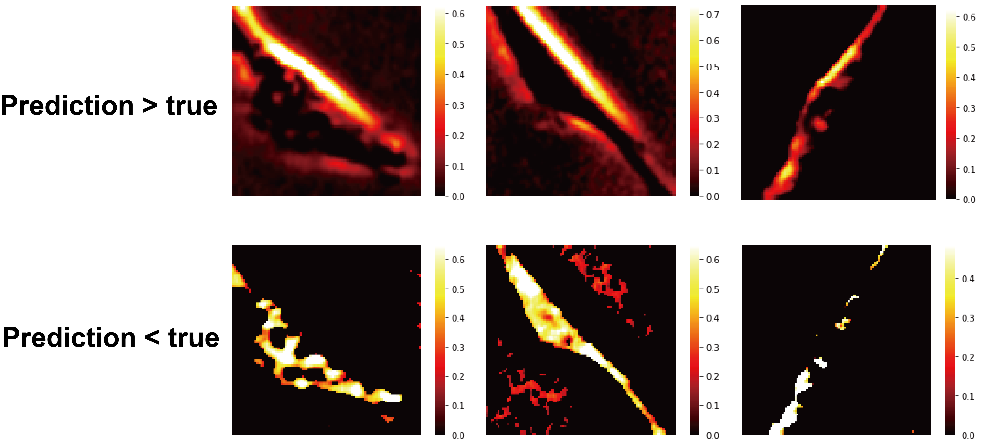


Fig. S9. Modified Grad-CAM visualization for the important regions that the regression model used for the prediction of MSC stiffness.


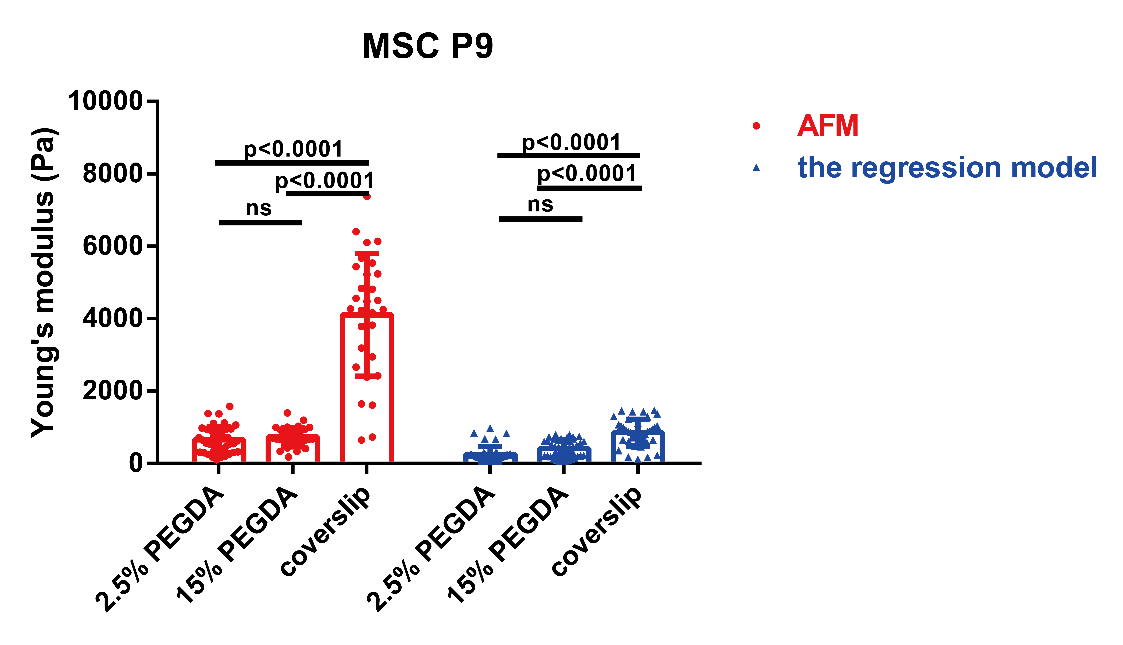


Fig. S10. Stiffness evaluation for MSC P9 cocultured with RAW264.7 and LSECs on substrates with different stiffness using AFM and the stiffness regression model.


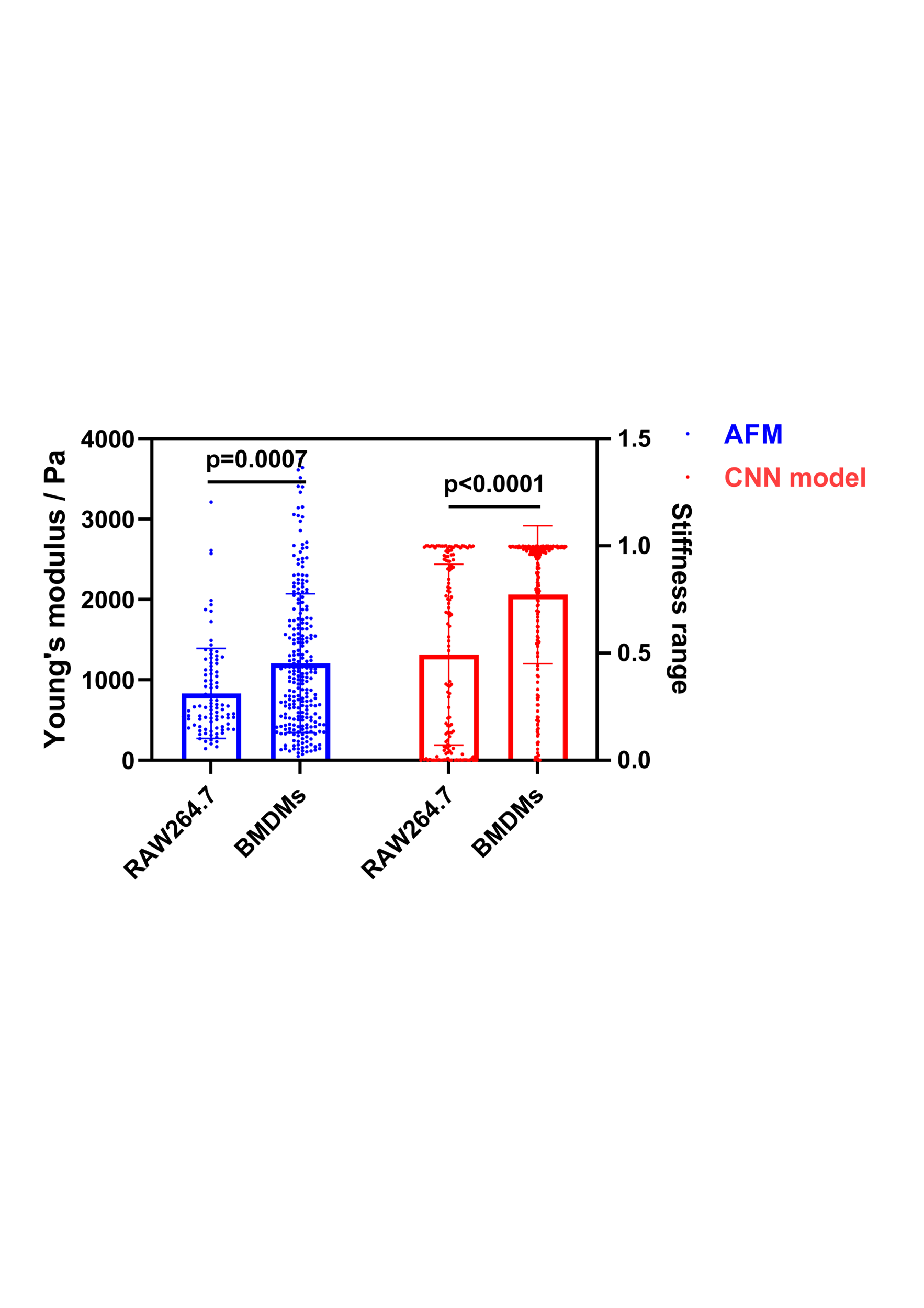


Fig. S11. Stiffness evaluation for RAW264.7 cell lines and BMDMs using AFM and the RAW264.7 stiffness classification model.


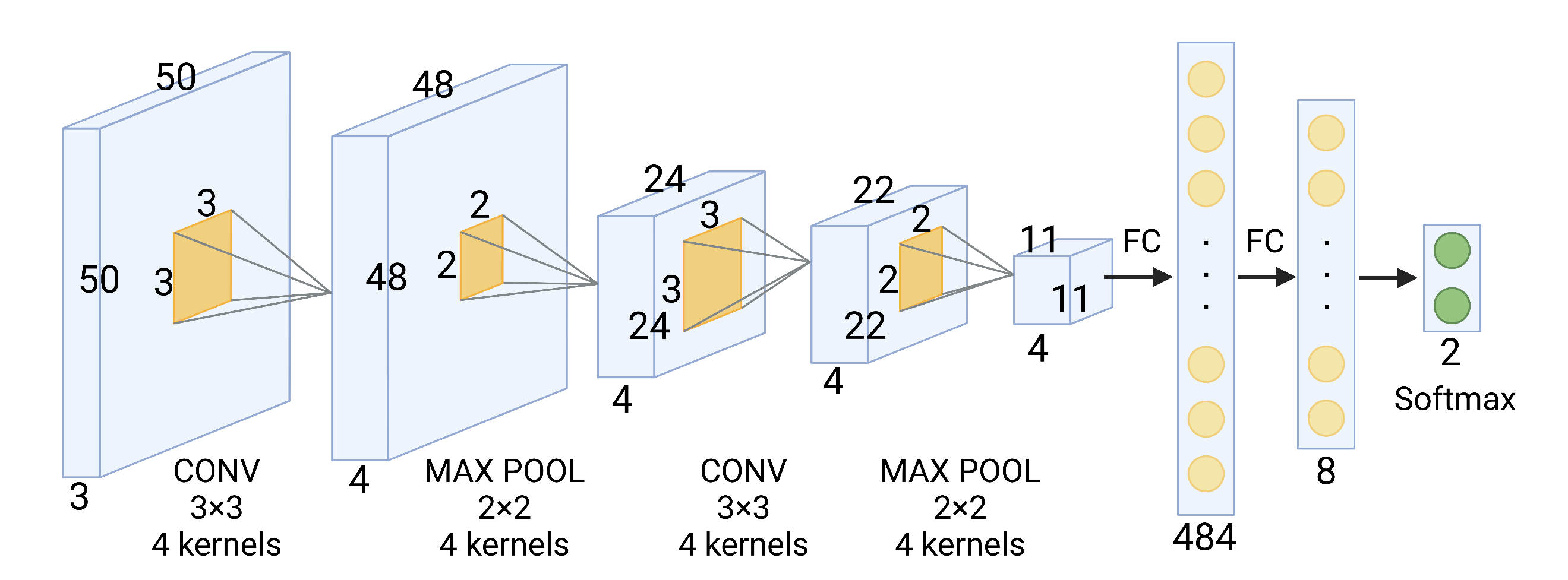


Fig. S12. The structure of stiffness classification model. The stiffness classification model was based on the CNN neural network, consisting of two 3×3 convolution layers, two 2×2 pooling layers, and two fully-connected layers. Rectified Linear Units (RLU) activation function was selected in the previous layers and softmax was selected as the activation function in the final layer. Adam algorithm as the optimizer and categorical_crossentropy as the loss function were chosen for model training with learning rates 1e-5.


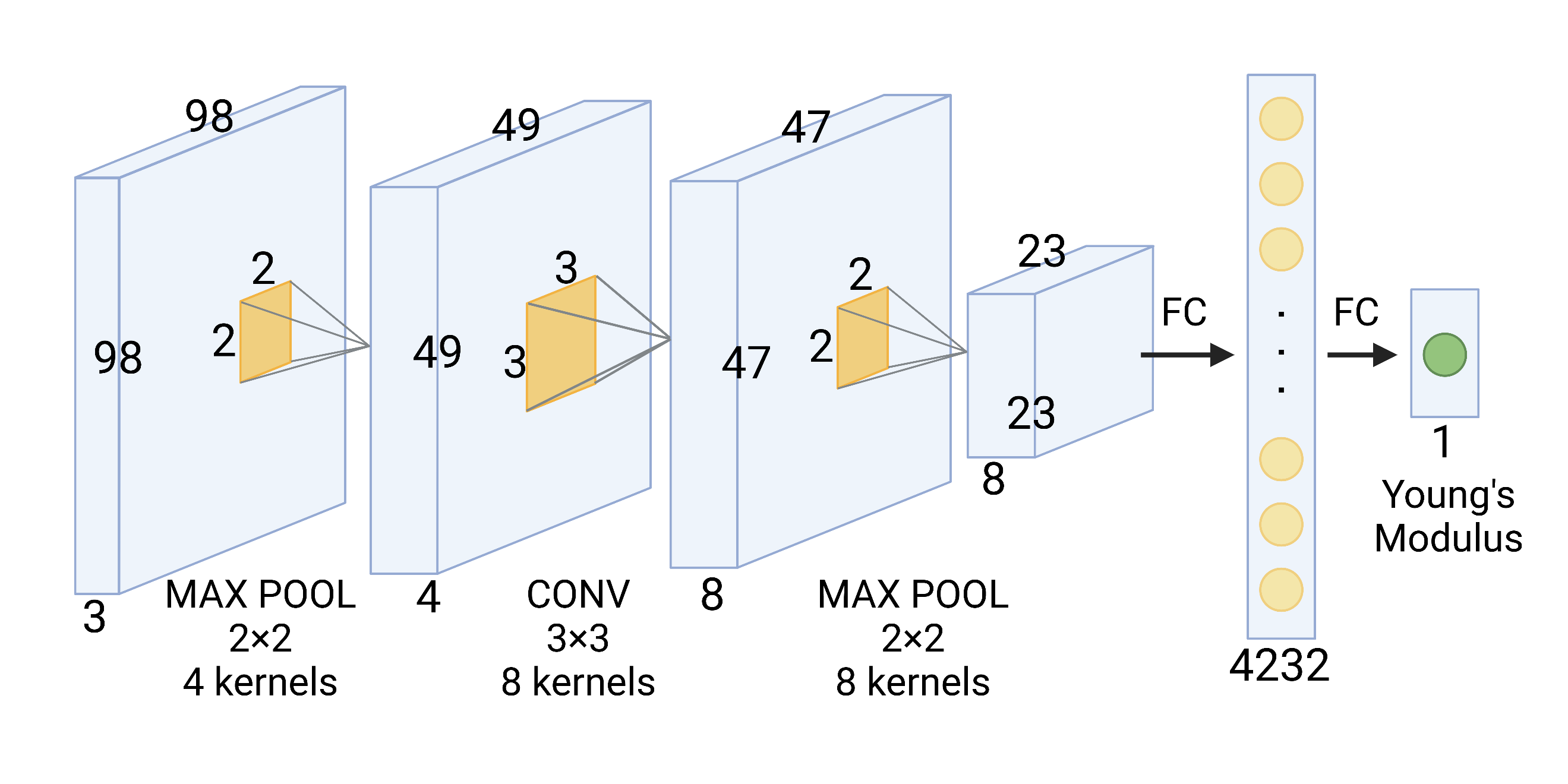


Fig. S13. The structure of stiffness regression model. The stiffness regression model was based on the CNN neural network, consisting of one 3×3 convolution layer, two 2×2 pooling layers, and two fully-connected layers. RLU activation function was selected in the previous layers and linear activation was selected as the activation function in the final layer. Adam algorithm as the optimizer and MSE as the loss function were chosen for model training with learning rates 1e-5.

Movie S1 (separate file). DC measurements for MSC stiffness.

Movie S2 (separate file). DC measurements for RAW264.7 stiffness.
